# Genome-wide analysis of a recently active retrotransposon, *Au* SINE, in wheat: content, distribution within subgenomes and chromosomes, and gene associations

**DOI:** 10.1007/s00299-017-2213-1

**Published:** 2017-11-21

**Authors:** Danielle Keidar, Chen Doron, Khalil Kashkush

**Affiliations:** Department of Life Sciences, Ben-Gurion University, Beer-Sheva, 84105 Israel

**Keywords:** Genome evolution, Transposable elements, SINE, Exonization, Wheat

## Abstract

****Key message**:**

**Here, we show that** ***Au*** **SINE elements have strong associations with protein-coding genes in wheat. Most importantly** ***Au*** **SINE insertion within introns causes allelic variation and might induce intron retention**.

**Abstract:**

The impact of transposable elements (TEs) on genome structure and function is intensively studied in eukaryotes, especially in plants where TEs can reach up to 90% of the genome in some cases, such as in wheat. Here, we have performed a genome-wide in-silico analysis using the updated publicly available genome draft of bread wheat (*T. aestivum*), in addition to the updated genome drafts of the diploid donor species, *T. urartu* and *Ae. tauschii*, to retrieve and analyze a non-LTR retrotransposon family, termed *Au* SINE, which was found to be widespread in plant species. Then, we have performed site-specific PCR and realtime RT-PCR analyses to assess the possible impact of *Au* SINE on gene structure and function. To this end, we retrieved 133, 180 and 1886 intact *Au* SINE insertions from *T. urartu, Ae. tauschii* and *T. aestivum* genome drafts, respectively. The 1886 *Au* SINE insertions were distributed in the seven homoeologous chromosomes of *T. aestivum*, while ~ 67% of the insertions were associated with genes. Detailed analysis of 40 genes harboring Au SINE revealed allelic variation of those genes in the *Triticum–Aegilops* genus. In addition, expression analysis revealed that both regular transcripts and alternative *Au* SINE-containing transcripts were simultaneously amplified in the same tissue, indicating retention of *Au* SINE-containing introns. Analysis of the wheat transcriptome revealed that hundreds of protein-coding genes harbor Au SINE in at least one of their mature splice variants. *Au* SINE might play a prominent role in speciation by creating transcriptome variation.

**Electronic supplementary material:**

The online version of this article (10.1007/s00299-017-2213-1) contains supplementary material, which is available to authorized users.

## Introduction

Transposable elements (TEs) make up a large fraction of plant genomes (Kidwell 2002), as they can reach up to 90% of the wheat genome (Charles et al. 2008). Retrotransposons are the most abundant class of TEs in plants (Kejnovsky et al. 2012; Kumar and Bennetzen 1999); they are divided into LTR retrotransposons and non-LTR retrotransposons, the latter of which include Long INterspersed Nuclear Elements (LINEs) and Short INterspersed Nuclear Elements (SINEs). SINEs are miniature elements (80–500 bp) that probably originated from an accidental retroposition of polymerase III-derived (pol III, e.g. tRNAs) transcripts (Wicker et al. 2007). Their 5′ region harbors an internal pol III promoter [composed of A and B boxes that are recognized by RNA polymerase III (Arnaud et al. 2001)], a family unique internal region (sized 50–200 bp), and a 3′ region. Their 3′ region can be either AT or A rich and it contains short tandem repeats (3–5 bp) or a poly(A) tail. SINE superfamilies (tRNA, 7SL RNA and 5S RNA) are defined by conserved pol III promoters. SINEs are non-autonomous, as they are only capable of transposition using proteins encoded by LINEs elements, while creating TSDs (5–15 bp) (Wicker et al. 2007). Several SINE families have been discovered in plants, such as in *Brassica napus* (Deragon et al. 1994) *Oryza sativa* (Hirano et al. 1994), *Nicotiana tabacum* (Yoshioka et al. 1993), *Myotis daubentonii* (Borodulina and Kramerov 1999) and others (Deragon and Zhang 2006; Wenke et al. 2011). While SINEs are less abundant in grasses compared to LTR retrotransposons (Kumar and Bennetzen 1999; Sabot et al. 2004), a SINE family termed *Au* SINE, discovered in high copy numbers in wheat (Ben-David et al. 2013; Yasui et al. 2001) was found to be widely distributed in higher plants (Fawcett et al. 2006; Yagi et al. 2011). The impact of SINEs on plant genomes is poorly studied, while it has been well studied in mammalians, e.g., MIR and *Alu* elements (Deininger and Batzer 1999; Lev-Maor et al. 2003; Makalowski 2003; Schmid 1998; Schmitz and Brosius 2011; Smit 1996, 1999).

Both MIR and *Alu* elements were found to play a role in the exonization process of protein-coding genes (Schmitz and Brosius 2011). In the exonization process, non-protein-coding sequences, primarily introns, become part of the mature RNA, creating alternative splice variants (Clavijo et al. 2017). It has been reported that fragments of *Alu* sequences, which exist in ~ 1.4 million copies in the human genome, may appear in the protein-coding region of mature RNAs (Makałowski et al. 1994; Nekrutenko and Li 2001). Nearly 1800 retrotransposon-derived exons were found in humans, mostly *Alu*-containing transcripts (Schmitz and Brosius 2011). However, the frequency of *Alu*-containing transcripts was found to be much lower than the alternatively spliced exons that do not contain an *Alu* sequence (Schmitz and Brosius 2011). In most cases, the insertion of *Alu* into the coding regions of mRNAs creates frame-shifts or premature termination codons, but sometimes it also creates new protein functions or had modified existing ones (Hilgard et al. 2002).

In a previous report, we showed that *Au* SINE retains retrotranspositional activity following allopolyploidization events in wheat (Ben-David et al. 2013). In this study, the availability of updated genome drafts for several wheat species, especially the genome draft and the RNA-seq database of bread wheat (*T. aestivum*) facilitated a genome-wide analysis of *Au* SINE in the wheat genome and transcriptome. We have retrieved *Au* SINE-containing sequences distributed among the seven homoeologous chromosomes of *T. aestivum*, and found strong association with hundreds of protein-coding genes; in most of our cases, *Au* SINE was found to be inserted within the introns of a gene. We then analyzed the impact of *Au* SINE on the structure of genes and found allelic variations of many genes, based on insertional polymorphism of *Au* SINE in various wheat species. Expression analysis of several genes by real-time RT-PCR, revealed that *Au* SINE might undergo exonization in *T. aestivum*. Genome-wide, *in-silico* analysis of the *T. aestivum* transcriptome revealed that tens of protein-coding genes harbor *Au* SINE in their coding sequence. Detailed analysis of 83 genes showed that at least 50 of them showed splice variants including or excluding an *Au* SINE. The possible impact of *Au* SINE on gene structure and function is discussed.

## Results and discussion

### Genome-wide analysis of *Au* SINE in genome drafts of *T. urartu, Ae. tauschii* and *T. aestivum*

The publicly available sequence drafts for *T. urartu, Ae. tauschii* and *T. aestivum* facilitated a genome-wide analysis, including: copy numbers, insertion sites and distribution of *Au* SINE elements in bread wheat and its diploid ancestors. The relatively short sequence (181 bp) (Deragon and Zhang 2006; Yasui et al. 2001) of *Au* SINE allowed us to identify and characterize intact elements together with their insertion sites. In addition, the updated genome draft sequence of *T. aestivum* was published for each chromosome separately, which allowed the analysis of *Au* SINE content in each one of the three subgenomes (A, B and D), and analysis of the distribution of *Au* SINE in the seven homoeologous chromosomes. To this end, using the MITE analysis kit (MAK) (Yang and Hall 2003b), we have retrieved 133, 180 and 1886 intact *Au* SINE insertions from *T. urartu, Ae. tauschii* and *T. aestivum* genome drafts, respectively. The copy number of *Au* SINE in the allohexaploid *T. aestivum* genome was ~ tenfold its copy number in the diploid genomes, *T. urartu* and *Ae. tauschii*, indicating the massive retrotransposition burst of *Au* SINE following allopolyploidization events; most probably, the retrotransposition burst occurred following allotetraploidization because a similar content of *Au* SINE was found in the genome draft of *Triticum turgidum* ssp. *dicoccoides* (data not shown). This finding provides additional evidence for our previous report, wherein, using real-time quantitative PCR analysis, we found that the content of *Au* SINE was up to tenfold higher in allopolyploid wheat species compared to diploid species (Yaakov et al. 2013b).

**Fig. 1:**
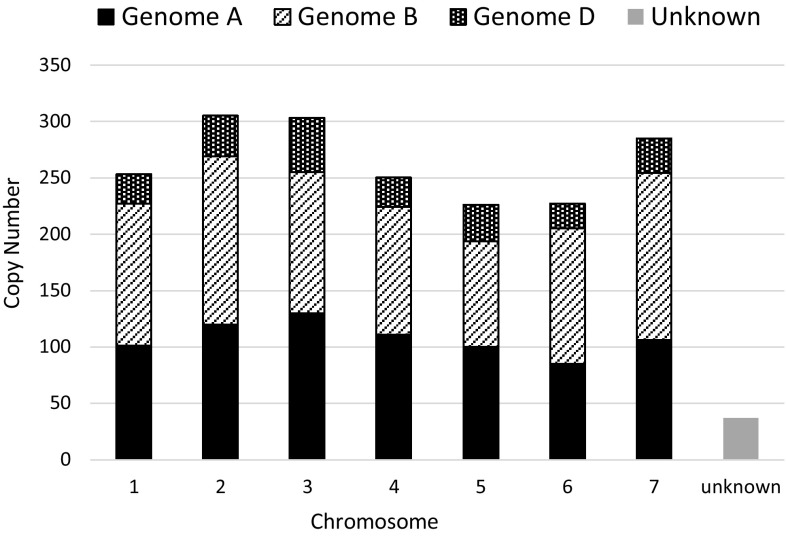
Copy number and distribution of *Au* SINE in *T. aestivum* (genome composition AABBDD) genome. Each chromosome (1–7) is defined by its genome composition (AA, BB and DD subgenomes). A total of 1886 *Au* SINE insertions were retrieved from the *T. aestivum* genome draft, while 753, 875, and 221 insertions were retrieved from A, B and D subgenomes, respectively. Note that 37 insertions were not mapped in the seven homeologous chromosomes and they are indicated as “unknown”

Of the 1886 retrieved *Au* SINE insertions from the *T. aestivum* genome, 1849 were mapped to the seven homoeologous chromosomes (Fig. 1), distributed among the seven chromosomes of the three subgenomes: AA, BB and DD. The copy number of *Au* SINE in the AA subgenome was ~ fivefold higher than its copy number in the diploid AA genome (753 vs. 133, respectively), indicating proliferation of the element in the AA genome following allopolyploidization. In addition, the copy number of *Au* SINE in the DD subgenome was nearly similar to its copy number in the DD diploid genome (221 vs. 180, respectively), indicating a lack of proliferation in the DD subgenome following allohexaploidization, and thus the retrotransposition burst of *Au* SINE might occurred at the allotetraploid level, around 0.5 million years ago (Feldman and Levy 2005) Our data strongly indicate that although *Au* SINE is an ancient retrotransposon family (arising prior to the divergence of monocots and eudicots), found in many groups of higher plants (Fawcett et al. 2006; Yagi et al. 2011), it retained retrotranspositional activity in the *Triticum–Aegilops* genus.

Analysis of the common insertions of *Au* SINE among the three genome drafts: *T. urartu, Ae. tauschii* and *T. aestivum* revealed that only 24 of the 2199 total insertions are common (monomorphic insertions in the three species), indicating the massive proliferation of *Au* SINE after the divergence of the diploid species, around 4 million years ago (Feldman and Levy 2005). Note that the analysis was done based on 100% identity (*e* value = 0) of the insertions sites (*Au* SINE-flanking sequences), as such the results might be underestimated. In addition, 124 (53.1%) of the 221 insertions in the DD subgenome of *T. aestivum* are in common with *Ae. tauschii*, while only 77 (10.2%) of the 753 insertions in the AA subgenome of *T. aestivum* are in common with *T. urartu* (the diploid donor of genome AA). This finding supports our above conclusion that the retrotransposition burst of *Au* SINE might have occurred at the allotetraploidization level in the AA and BB subgenomes.

In a previous study (Ben-David et al. 2013), we reported on ~ 38% of the 3706 retrieved *Au* SINE insertions from the publicly available unassembled 454 pyrosequencing of *T. aestivum* were in transcribed regions. Here, we report on nearly half of the number of *Au* SINE insertions (1886) in the assembled and sorted genome draft of *T. aestivum* [(Clavijo et al. 2017) (http://www.ebi.ac.uk/ena/data/view/GCA_900067645.1, plants.ensembl.org/Triticum_aestivum/)], indicating the sequence redundancy in the 454 pyrosequencing data (Brenchley et al. 2012), as we have noted in our previous study (Ben-David et al. 2013). Annotation of the 1886 *Au* SINE insertion sites revealed that 1268 (67.2%) insertions were located within or near (up to 500 bp upstream or downstream) the DNA sequence of predicted protein-coding genes, 213 (11.2%) insertions within non-coding RNA (ncRNA) sequences, 253 (13.4%) insertions within other class I (173 insertions) and class II (80 insertions) TEs, and the remaining 152 *Au* SINE insertions were in non-coding DNA sequences. The data demonstrate that ~ 78.5% of the *Au* SINE insertions are in transcribed sequences (excluding insertions in other TEs), which might indicate a strong association of *Au* SINE with genes. Protein-coding genes that harbor *Au* SINE include: *Transcription factors, Zinc finger-containing proteins, Homeobox genes, Methyltransferase, RNA-directed DNA polymerase, DNA-damage–repair, Ethylene-forming-enzyme-like dioxygenase, chromatin-associated protein, WRKY transcription factor*, and others (Table S1).

### Allelic variation in protein-coding genes caused by *Au* SINE

**Fig. 2:**
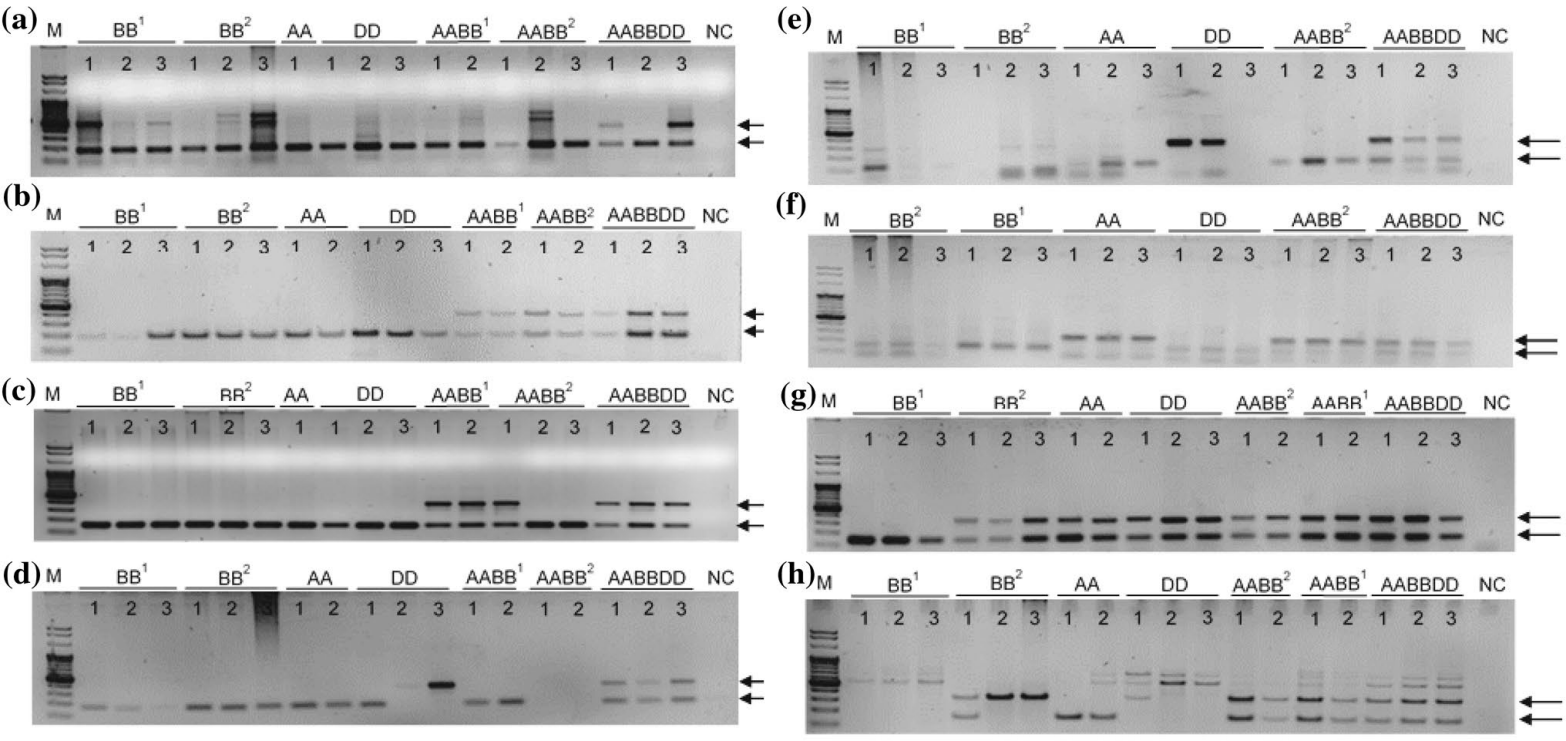
Site-specific PCR analysis using primers from *Au* SINE-flanking sequences. In each panel, the upper arrow represents a “full site” and the lower arrow represents an “empty site”. “M” represents the size marker in all the gels, “NC” represents for negative control, ddH_2_0 was used as template in PCR reactions. The PCR analysis was performed in accessions of: BB^1^ = *Ae. searsii*, BB^2^ = *Ae. speltoides*, AA = *T. urartu*, DD = *Ae. tauschii*, AABB^1^ = *T. durum*, AABB^2^ = *T. dicoccoides*, AABBDD = *T. aestivum*. Note that for all polymorphic *Au* SINE insertions the difference between the “full site” fragment and the “empty site” is ~ 181 bp, the size of *Au* SINE. Numbers above each lane represent the genomic replicates. **a** Au SINE insertion in *Putative Serine*/*threonine-protein kinase* (case 8 in Table 1). The “full site” is 399 bp and the “empty site” is 218 bp. The insertion is unique to *T. aestivum* (amplified in two accessions 1 and 3). Note that the rest of the upper bands are non-specific PCR products as seen by sequence validation. **b** *Au* SINE insertion in an Predicted protein (case 22 in Table 1). The “full site” is 395 bp and the “empty site” is 214 bp. The insertion was seen in accessions of *T. durum, T. dicoccoides* and *T. aestivum*. **c** *Au* SINE insertion in *Putative ATP-dependent RNA helicase DHX36* (cases 13 in Table 1). The “full site” is 387 bp and the “empty site” is 208 bp. The insertion was seen in *T. durum, T. dicoccoides* and *T. aestivum*. **d** *Au* SINE insertion in *Inositol hexakisphosphate and diphosphoinositol-pentakisphosphate kinase* (cases 27 in Table 1). The “full site” is 377 bp and the “empty site” is 196 bp. The insertion was seen in *Ae. tauschii* and *T. aestivum*. **e** *Au* SINE insertion in *Chloride channel protein CLC-c* (cases 25 in Table 1). The “full site” is 323 bp, and the “empty site” is140bp. The insertion was seen in *Ae. tauschii* and *T. aestivum*. **f** *Au* SINE insertion in an *Predicted protein* (case 31 in Table 1). The “full site” is 274 bp and the “empty site” is 97 bp. The insertion was seen in *T. urartu, T. dicoccoides* and *T. aestivum*. **g** *Au* SINE insertion in *SIN3 transcription regulator family member B* (cases 40 in Table 1). The “full site” is 342 bp and the “empty site” is 165. The insertion was seen in all tested species except for *Ae. searsii* accessions. **h** *Au* SINE insertion in *Calcineurin-like metallo-phosphoesterase* (cases 36 in Table 1). The “full site” is 352 bp and the “empty site” is 180 bp. The insertion was seen in *Ae. speltoides, T. durum, T. dicoccoides* and *T. aestivum*

To examine whether *Au* SINE insertions into protein-coding genes might cause allelic variation (based on presence/absence of the element) among species in the *Aegilops–Triticum* genus, we have performed site-specific PCR analysis to amplify *Au* SINE elements within genes in *Aegilops* and *Triticum* species (see plant material), including diploid (AA, BB and DD genome species), tetraploid (wild emmer and durum, AABB genome), and hexaploid (AABBDD genome) species. Primers were designed from *Au* SINE-flanking sequences; so in each case, a larger PCR product represents a full site (presence of an *Au* SINE in the gene), while a smaller PCR product represents an empty site (absence of an *Au* SINE in the gene) (see Fig. 2). Note that in most cases PCR products were sequenced for validation of the presence/absence of *Au* SINE. To this end, 40 arbitrarily selected genes harboring *Au* SINE in *T. aestivum* were analyzed by site-specific PCR for presence/absence of the element in the genome of other wheat species (Table 1, supplementary Figure S1). Monomorphic *Au* SINE insertions in all the tested *Aegilops* and *Triticum* species were seen in 6 of the 40 analyzed genes (cases 1–6 in Table 1, see an example in Figure S1a), indicating old insertions of *Au* SINE, most probably before the divergence of the *Aegilops* and *Triticum* species. For the remaining 34 genes, polymorphic *Au* SINE insertions in *Aegilops* and *Triticum* species were seen (Fig. 2 and Figure S1). We have classified the insertion patterns into two main classes: (1) *Au* SINE has inserted into a gene only in the allopolyploid species (Fig. 2a–c), either in the *T. aestivum* only (cases 7–8 in Table 1) or in both *T. turgidum* and *T. aestivum* (cases 9–24 in Table 1), indicating that these insertions occurred following allopolyploidization; (2) *Au* SINE insertion occurred in the diploid species and was further inherited to the derived polyploid species, either from the DD genome (*Ae. tauschii*) donor (cases 25–29 in Table 1; Fig. 2d, e), the AA genome (*T. urartu*) donor (cases 30–33 in Table 1; Fig. 2f), the BB genome (*Ae. speltoides* and/or *Ae. searsii*) donors (cases 34–36 in Table 1, Figure S1w), or the insertion was seen in different diploid donors (cases 37–40 in Table 1, Fig. 2g, h). Note that in some cases polymorphic insertions were seen among different accessions of the same species, creating genetic variation within the same species; for example, insertions in some accessions of *T. aestivum* (Fig. 2a) or in some accessions of *Ae. tauschii* (Fig. 2d, e). Additionally, we have used accessions of *Ae. speltoides* and *Ae. searsii* because they are considered the potential donors of BB genome to wheat (Feldman and Levy 2005; Yaakov et al. 2012). To this end, our data indicate the dynamic nature of *Au* SINE throughout wheat evolution and its strong association with genes that might impact gene structure and function.

**Table 1:** Site-specific PCR analysis of *Au* SINE insertional polymorphism within genes in *Triticum* and *Aegilops* species

No.	Gene accession number^a^	Gene product^b^	Primer R sequence^c^	Primer L sequence^c^	Location within gene^d^	Existence of *Au* SINE in the genome^e^					
						AA	BB^*^	BB^#^	DD	AABB	AABBDD
1	TRIAE_CS42_1DL_TGACv1_061151_AA0187150	DnaJ homolog subfamily C member 13	GAATTTAGTTTGCGGTTCCAAG	TGTGGTACATATCCATGCGTTT	Intron	√	√	√	√	√	√
2	TRIAE_CS42_2BL_TGACv1_129350_AA0379730	CLPTM1-like membrane protein cnrB	ACGAAGAACTAAAAGCCGTGAA	CTGCTATACCATGCGATCGTT	Intron	√	√	√	√	√	√
3	TRIAE_CS42_7BL_TGACv1_577050_AA1863990	Predicted protein	CGTATTCAAAGATGTTCCACGA	GGCTGGGTTAAGATTGTTTTTG	Exon	√	√	√	√	√	√
4	TRIAE_CS42_7BS_TGACv1_591977_AA1927210	Predicted protein	ATGAAGACAACAAGTGCCACAC	GAATAAACATGCCATTCTGCAA	100 bp downstream	√	√	√	√	√	√
5	TRIAE_CS42_4DS_TGACv1_363130_AA1183390	Rht-D1b	TTGCATCAACTCACCATGAAAT	ATGTGTGAACCGACAACTGAAG	Intron	√	√	√	√	√	√
6	TRIAE_CS42_4DL_TGACv1_342699_AA1119920	Superoxide dismutase [Cu–Zn] 3	GCGACACACCAAAAATTTCATA	GCACCACCTGCTGATACACTTA	Intron	√	√	√	√	√	√
7	TRIAE_CS42_U_TGACv1_640941_AA2079970	Disease resistance protein RPP13-like	TTACTGGGACCTTCCACACC	GCCATCCATTTCCATTTCAG	Exon	×	×	×	×	×	√
8	TRIAE_CS42_1BS_TGACv1_050314_AA0170550	Putative Serine/threonine-protein kinase CBK1	ACATGGATGAGCAGGACTAGGT	TCAGAGGGGTCAGGAATAGAAA	Intron	×	×	×	×	×	√
9	TRIAE_CS42_7BL_TGACv1_577920_AA1886220	Zinc transporter ten-like	CCCAAAGATCGCTAGATCAGA	TGTTCAAACACGGGGATGTA	Intron	×	×	×	×	√	√
10	TRIAE_CS42_1BL_TGACv1_032021_AA0124300	Putative E3 ubiquitin-protein ligase HERC1	TCCTTCTCAGGGCGTAGAAA	TTGGTTTACATTCACAGGATCAA	Intron	×	×	×	×	√	√
11	TRIAE_CS42_1BS_TGACv1_049809_AA0161910	Predicted protein	TGTCTGGTGCTTGTGAAGAAAC	ATCGAATCACATCCCTTTCAGT	Intron	×	×	×	×	√	√
12	TRIAE_CS42_6BL_TGACv1_499645_AA1588080	Rho guanine nucleotide exchange factor 8	TCCTTTCCTACCCACAGATCAT	CGCAGACTGATTCCCTGTCTAT	Intron	×	×	×	×	√	√
13	TRIAE_CS42_5AL_TGACv1_377324_AA1245930	Putative ATP-dependent RNA helicase DHX36	AGCATTGGGGAGTTTCTATCAG	TAAGAGCCCAACAAATGTCAAA	Intron	×	×	×	×	√	√
14	TRIAE_CS42_5BL_TGACv1_406235_AA1342580	Predicted protein	TGAGTGGCAAAACTCTCAGATG	GCCTACATCGACCAAATTCTTC	Intron	×	×	×	×	√	√
15	TRIAE_CS42_2AL_TGACv1_093126_AA0272720	Cell division protein FtsZ homolog 1, chloroplastic	AGTGCCTGACGTGGTAAGAAAT	GAATTTCTGTTTGCAGTGCTTG	Intron	×	×	×	×	√	√
16	TRIAE_CS42_2BL_TGACv1_130367_AA0409600	Exocyst complex component 2	GTGAGAACTGAGCATGAACTGG	ATCCATTAGGCCTTGGGTAACT	Intron	×	×	×	×	√	√
17	TRIAE_CS42_3B_TGACv1_220590_AA0709880	Josephin family protein	CAGCTGTACACTTCAAACCAATG	TATGATTTGATCCGAAATGCAA	Intron	×	×	×	×	√	√
18	TRIAE_CS42_1BS_TGACv1_049553_AA0156720	Predicted protein	GCTATCGCCTGGTTATGAGTTC	AAGAGGATCATTTGCTTTTCCA	Exon/Intron	×	×	×	×	√	√
19	TRIAE_CS42_2BS_TGACv1_146572_AA0468420	2-dehydro-3-deoxyphosphooctonate aldolase	GCAGACATTTTTGCTCAACCTT	CATGATGATTCCCTTGATGTTG	Intron	×	×	×	×	√	√
20	TRIAE_CS42_2AL_TGACv1_096183_AA0317680	ATP-dependent RNA helicase SUPV3L1, mitochondrial	ATCTACGCCTTATTTGCTCTGG	GGTAAAGTGTGCCTTTTTGAGG	Intron	×	×	×	×	√	√
21	TRIAE_CS42_2BL_TGACv1_129880_AA0398630	Oligoribonuclease	TGCTAGTGGACTCAACCAAATC	TGGCCTCAGAGCCTAGTAACA	Intron	×	×	×	×	√	√
22	TRIAE_CS42_1AL_TGACv1_000103_AA0003430	Predicted protein	CAGACCACAATGGGTATGGTTA	CCATAGAACTCCATCAACATCG	intron	×	×	×	×	√	√
23	TRIAE_CS42_5BL_TGACv1_405351_AA1325650	MADS-box transcription factor 14	CTAGGTCCATCTGGTCCCTAAAG	CTTTGACTACCCCACATTAGCTG	Intron	×	×	×	×	√	√
24	TRIAE_CS42_5BL_TGACv1_404700_AA1308770	E3 ubiquitin-protein ligase AIP2	AGATTTGTGATAGCAGCACCAG	TTGTGAGTTACCTTGAGCCTAGC	Intron	×	×	×	×	√	NA
25	TRIAE_CS42_2DL_TGACv1_158055_AA0507480	Chloride channel protein CLC-c	CAGCTGTGCTGATTTGCCTA	GAAAGGATGATGCAGAGTTTCA	Intron	×	×	×	√	×	√
26	TRIAE_CS42_2DS_TGACv1_177403_AA0575920	5′–3′ exoribonuclease 3	CATGGCATAACATCGAACAAAA	AGCCGCCTTTTTGAATTTTACT	Intron	×	×	×	√	×	√
27	TRIAE_CS42_4DS_TGACv1_361106_AA1161070	Inositol hexakisphosphate and diphosphoinositol-pentakisphosphate kinase 1	TCTCCCTCACAGATCACTTCAA	TTAGCCACAAGTTTGGATAGGC	Intron	×	×	×	√	×	√
28	TRIAE_CS42_4DL_TGACv1_343519_AA1135850	Conserved oligomeric Golgi complex subunit 6-like	GTGCCAAGTGAATGAAGTCAAG	CTGACACCATGGTACCCTAACA	Intron	×	×	×	√	×	√
29	TRIAE_CS42_6DL_TGACv1_526989_AA1696800	Serine/threonine-protein kinase	TAGTCTCTAATTTGCGGGTCCA	CACGCATGTCACCAAACATTA	Intron	×	×	×	√	×	√
30	TRIAE_CS42_4AL_TGACv1_290382_AA0984800	ERBB-3 BINDING PROTEIN 1	TACCGTCAGCAGAACACCAC	TGAGATGTGTGAGAAGGGTGA	Intron	√	×	×	×	√	√
31	TRIAE_CS42_5AL_TGACv1_378959_AA1255190	Leucine zipper protein	AGATTGCTGGAAAATAAGGACAA	TTTTGGATCGTGCCTAGGAG	Intron	√	×	×	×	√	√
32	TRIAE_CS42_2AL_TGACv1_093836_AA0287830	Putative pectinesterase/ pectinesterase inhibitor 51	TCTCCCTTTGTCACTTTTGCTT	GAAGCATCTTTCTGCCATCTTT	Intron	√	×	×	×	√	√
33	TRIAE_CS42_3AS_TGACv1_211370_AA0689310	C3H2C3 RING-finger protein	CCTCAGGAGGTGAATTGCTC	TTAACCCGCCTCACTTTGTC	Exon/Intron	√	×	×	×	√	NA
34	TRIAE_CS42_7AL_TGACv1_558277_AA1791890	ELAV-like protein 1	ATAGCCAGTGGTAGGCCACA	ACACTGACCGGATTTGAACC	Intron	×	√	√	×	√	√
35	TRIAE_CS42_2BS_TGACv1_145935_AA0449980	Mediator of RNA polymerase II transcription subunit 14-like	CCCAAAAGATGAAATAGCAACC	GAAGAGGAAGGGCCAGTATGTT	Intron	×	×	√	×	√	√
36	TRIAE_CS42_5BL_TGACv1_407697_AA1358910	Calcineurin-like metallo-phosphoesterase superfamily protein	GTACATCAGTAGCGCAATGGAA	GAACTCCCAAGAAGAGGACCTT	intron	×	×	√	×	√	√
37	TRIAE_CS42_4AL_TGACv1_288293_AA0943850	Rho GTPase-activating protein 7	CCCTCAAATGCAAAGCGTAT	CTCCTCATGCTACGACGACA	Intron	√	√	×	√	√	√
38	TRIAE_CS42_7DS_TGACv1_624740_AA2063030	Kanadaptin	CATGTGTGCTTCCAAGATCG	ACCATGACTGGAATCGAAGG	Intron	√	×	√	√	√	√
39	TRIAE_CS42_4DL_TGACv1_342534_AA1116030	Serine-aspartate repeat-containing protein I-like	TGGAACCTGTCGGCTCTATTAT	AAGAATGATGGTCATGGATGTG	Intron	×	√	√	√	√	√
40	TRIAE_CS42_1BS_TGACv1_049347_AA0149810	SIN3 transcription regulator family member B	ACACGGCATATGGGTAATTGA	ATTCTGGCCACGTGATCTCTAT	Intron	√	×	√	√	√	√

^a^Gene accessions from *EnsemblPlants* (http://plants.ensembl.org/Triticum_aestivum)

^b^Based on gene annotation from *EnsemblPlants* with *e* value < 10^−10^.

^c^Primers were designed from *Au* SINE-flanking sequences.

“NA (Not available)” represents monomorphic cases in which the original genome that displays *Au* SINE insertion in the gene cannot be determined

^d^Location of *Au* SINE within or adjacent the gene: exon, intron, exon/intron junction or downstream of the gene

^e^AA = *T. urartu*, BB^*^ = *Ae. searsii*, BB^#^ = *Ae. speltoides*, DD = *Ae. tauschii*, AABB = *T. turgidum*, AABBDD = *T. aestivum*, “√"-full site in at least one accession of the same species; “×" empty site in all accessions of the same species, *NA* data not available

### Expression analysis of protein-coding genes harboring *Au* SINE

**Fig. 3:**
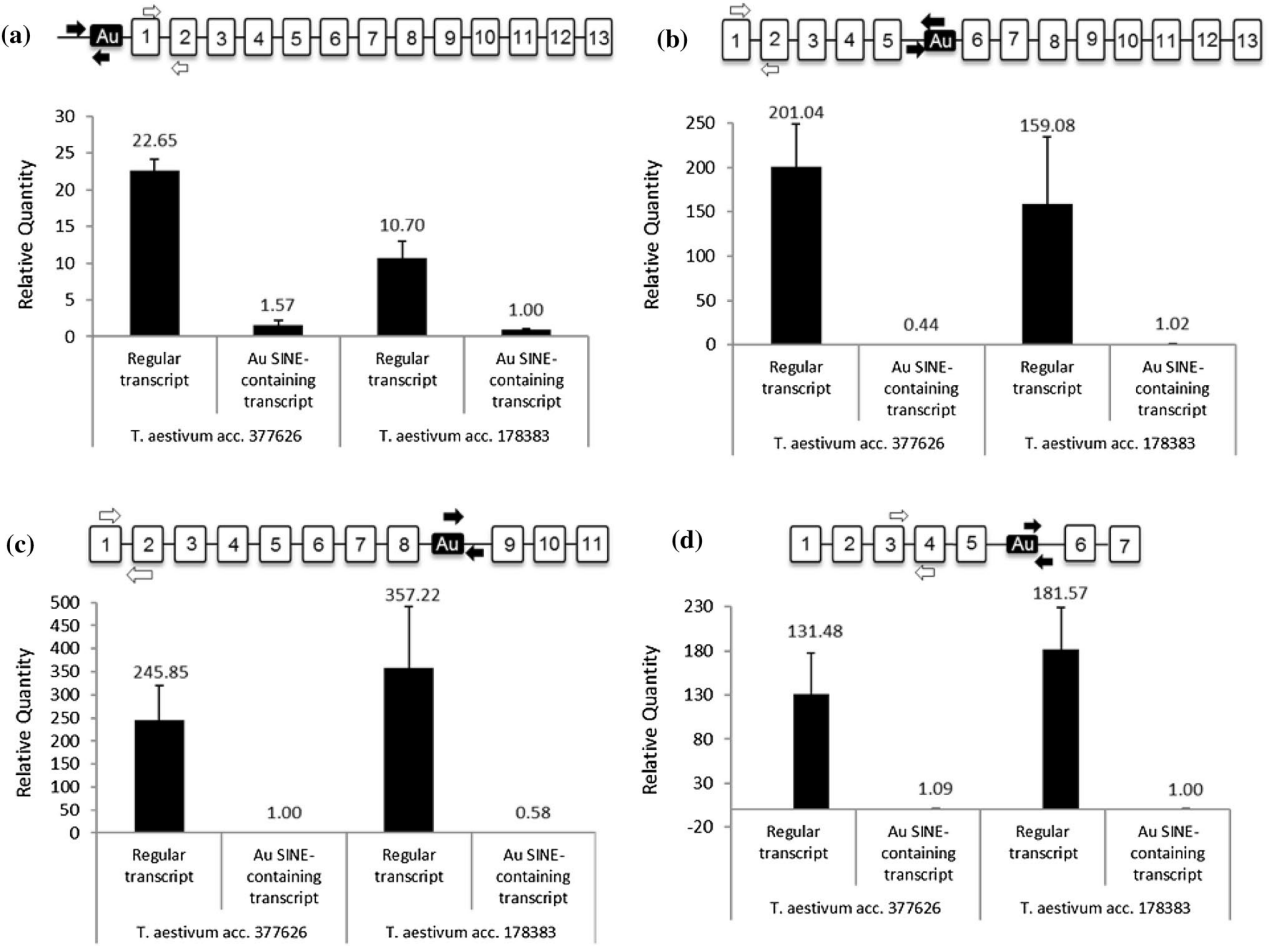
Relative expression levels of genes harboring *Au* SINE in two accessions of *T. aestivum*, as seen by realtime RT-PCR analysis. In each accession, the “regular transcript” compared to the *Au* SINE-containing transcript were analyzed. On top of each panel, a scheme of the analyzed gene, while the numbered boxes the exons and the black boxes note the *Au* SINE insertion. White arrows note the primers used to amplify the “regular transcript”, while the black arrows note the primers used to amplify the *Au* SINE-containing transcript. Expression levels (*Y* axis) were measured relative to *ACTIN*, and the exact relative expression (fold) is indicated by on top of each bar. Standard error on top of each bar was measured using three biological replicates. All the analyzed genes display the same trend of significantly higher expression levels of the regular transcript compared to the *Au* SINE-containing transcript, in the two tested *T. aestivum* accessions. The 4 analyzed genes: **a** *DnaJ homolog* (case 1 in Table 1), **b** *Calcineurin-like metallo-phosphoesterase* (case 36 in Table 1), **c** *Putative Serine*/*threonine-protein kinase* (case 8 in Table 1), and **d** *Superoxide dismutase* (case 6 in Table 1)

Sequence analysis of the 40 genes (Table 1) revealed that in most cases (35 of the 40) *Au* SINE had inserted in the intron region of the gene, indicating that *Au* SINE might be spliced out in the mature transcripts. In 4 cases (cases 3, 7, 18 and 33), the *Au* SINE was found to be part of an exon region, indicating that it might have underwent exonization throughout wheat evolution. In one case (case 4), the insertion was found 100 bp downstream to the gene. The phenomenon of SINE exonization has been reported in several studies in humans and other primates (Lev-Maor et al. 2003; Makałowski et al. 1994; Nekrutenko and Li 2001; Sorek et al. 2002), but has not been reported previously in plants. Here, we have analyzed the expression of several genes harboring *Au* SINE insertions in *T. aestivum*, using realtime RT-PCR, and found that most of those genes are expressed in bread wheat. Note that we have used two sets of primers (Table S2) for the expression analysis; the first set was designed to amplify exon–exon junction rescripts, and the second set of primers was designed to amplify chimeric (*Au*-SINE/flanking) transcripts, if they exist (see schemes on top of each one of the four panels in Fig. 3). While for most genes the exon–exon junction transcripts were amplified, no chimeric transcripts were seen, indicating that the intron harboring *Au* SINE was spliced out in the mature RNA. However, the expression analysis of four genes revealed that both the regular transcript (based on exon–exon junction amplification) and the chimeric (*Au* SINE/flanking) were simultaneously amplified in the same tissue, indicating retention of *Au* SINE-containing intron (Fig. 3). Note that the purity of each cDNA sample was tested using site-specific PCR reaction with primers from two exons of *Actin* gene, giving different amplification products for cDNA and genomic DNA. No DNA contamination was detected (Figure S2). In addition, the melting curves of the 4 cases are presented in Figure S3.

The expression analysis of a gene (case 1 in Table 1) that codes for *DnaJ homolog* [considered as chaperones in eukaryotes (Westermann et al. 1996)], in two accessions of *T. aestivum* revealed that both the regular transcript (based on primers designed from exon 1-exon two junction), and the chimeric (*Au* SINE/flanking) were simultaneously amplified at different levels (Fig. 3a). The regular transcript level was ~ 22-fold higher compared to the chimeric transcript in one accession, and ~ 11-fold higher in the second accession. Note that the transcript levels were relative to *ACTIN* transcription and three biological replicates were used in each accession. The expression analysis of a gene (case 36 in Table 1) that codes for *Calcineurin-like metallo-phosphoesterase* [involved in phosphorylated proteins substrates, nucleic acids or phospholipids (KEPPETIPOLA and SHUMAN 2006)] revealed that both the regular and the chimeric transcripts were simultaneously amplified, while the level of the regular transcript was over 160 fold higher compared to the chimeric transcript in both *T. aestivum* accessions (Fig. 3b). The *Au* SINE inserted into the intron located between exon 5 and exon 6 of this gene. The expression analysis of a gene (case 8, Table 1) that codes for *Putative Serine*/*threonine-protein kinase* [belong to the family of transferases (Huala et al. 1997)] revealed that both the regular and the chimeric transcripts were simultaneously amplified, while the level of the regular transcript was over 250 fold higher compared to the chimeric transcript in both *T. aestivum* accessions (Fig. 3c). The *Au* SINE inserted into the intron located between exon 8 and exon 9 of this gene. Finally, the expression analysis of a gene (case 6 in Table 1) that codes for *Superoxide dismutase* [catalyzes the dismutation of the superoxide (Kliebenstein et al. 1998)] revealed that both the regular and the chimeric transcripts were simultaneously amplified, while the level of the regular transcript was over ~ 130 fold higher compared to the chimeric transcript in both *T. aestivum* accessions. In this case, the *Au* SINE had inserted into the intron located between exon 5 and exon 6 of this gene. The relatively very low expression of the *Au* SINE-containing transcripts might indicate that these alternative transcripts do not have a major impact on the normal function of the proteins, but they might lead to the creation of modified proteins with new functions, similarly as was reported in animal and human systems (Lev-Maor et al. 2003; Makałowski et al. 1994; Nekrutenko and Li 2001; Schmitz and Brosius 2011; Schwartz et al. 2009; Sorek et al. 2002).

### Genome-wide analysis of *Au* SINE-containing transcripts in *T. aestivum*

**Fig. 4:**
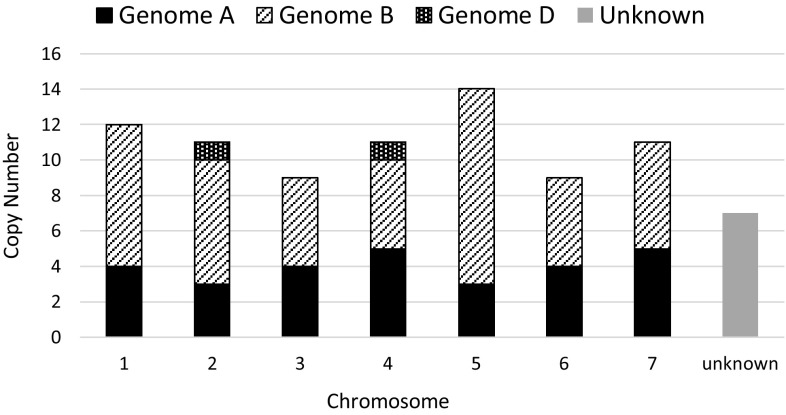
Distribution of 84 *Au* SINE-containing genes in the seven homoeologous chromosomes of *T. aestivum*. Each chromosome (1–7) is defined by its genome composition (AA, BB and DD subgenomes). A total of 28, 47 and 2 *Au* SINE-containing genes were retrieved from AA, BB and DD subgenomes, respectively. Note that 7 *Au* SINE-containing genes were not mapped in the seven homoeologous chromosomes and they are indicated as “unknown”

**Fig. 5:**
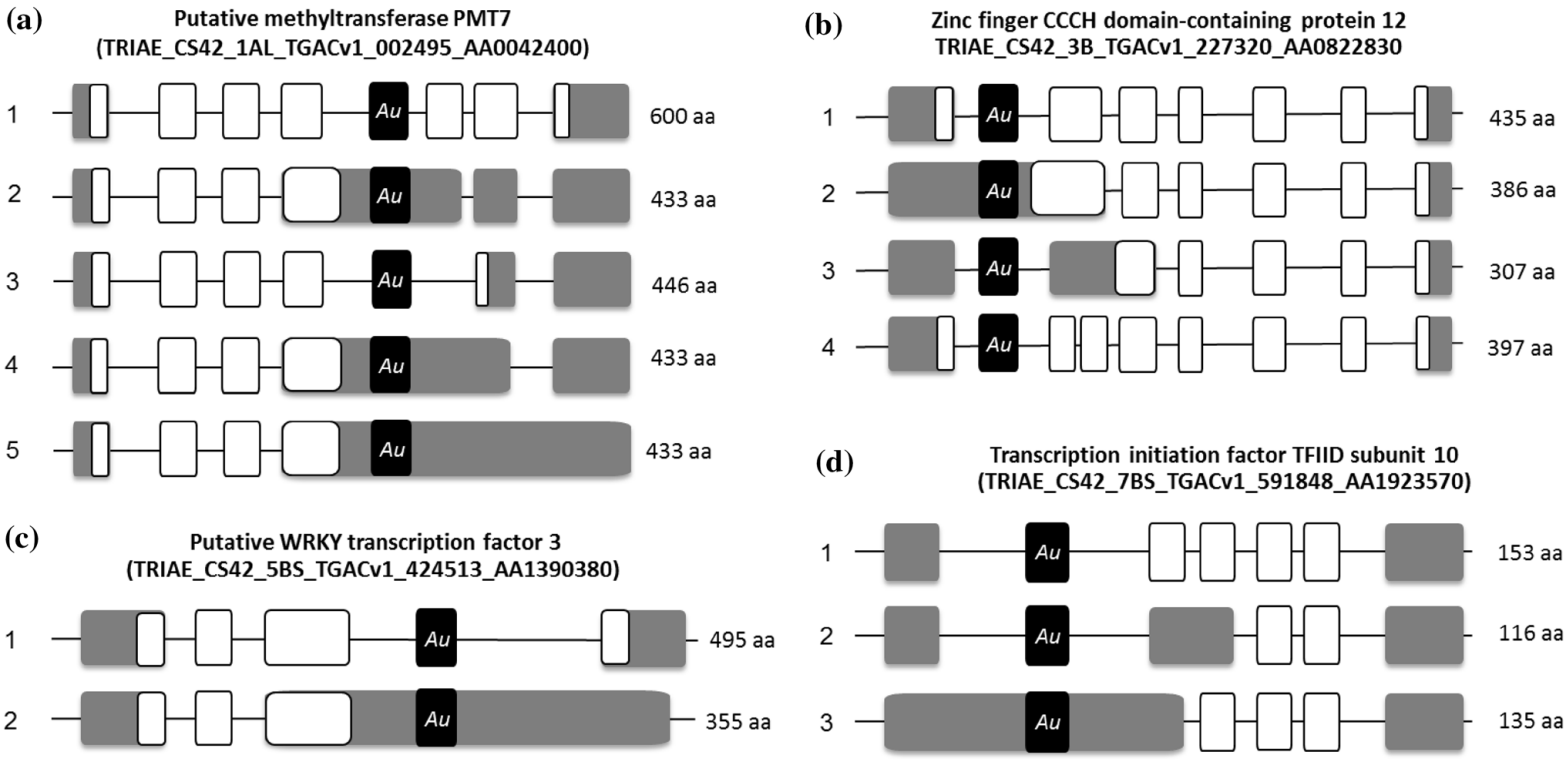
Splice variants (transcripts) of four *Au* SINE-harboring genes (**a**–**d**). The name of the gene and *EnsemblPlants* accessions number are indicated on top. Gray boxes represent exons and lines represent introns. White boxes represent CDS (coding sequences) regions. Note that the mature transcripts consist of exons only; thus, we kept here the intron regions to indicate the exact location of *Au* SINE (*black* boxes) in the mature transcript. The predicted protein for each splice variant is indicated on right. **a** Transcripts 2, 4 and 5 contain *Au* SINE. **b** Transcript 2 contains *Au* SINE. **c** Transcript 2 contains *Au* SINE. **d** Transcript 3 contains *Au* SINE

We have performed a genome-wide analysis of *Au* SINE-containing transcripts from the updated RNA-seq database of bread wheat (plants.ensembl.org/Triticum_aestivum/Info/Annotation/) to reveal exonization events of *Au* SINE. Using the MAK software, 113 *Au* SINE-containing transcripts (*Au* SINE and flanking sequences 500 bp upstream and downstream of the element) were retrieved from the *T. aestivum* transcriptome database. Detailed analysis of the 113 transcripts revealed that they belong to 83 protein-coding genes. Of the 83 genes that harbor *Au* SINE in their mature transcript, 76 were mapped in the seven homoeologous chromosomes, while 27, 47 and 2 were found in AA, BB and DD subgenomes, respectively (Fig. 4). Detailed analysis using the *EnsemblPlants* scripts revealed that 50 of the 83 genes showed different splice variants (Table 2), while many of those transcripts harbor *Au* SINE (Table 2). The number of splice variants for the 50 genes ranged between 2 and 9 transcripts, while at least 1 splice variant was an *Au* SINE-containing transcript (Table 2). For example: a gene that codes for *putative methyltransferase* (Table 2) showed 5 splice variants in chromosome 1A of *T. aestivum*, while 3 variants harbor *Au* SINE within their transcript (Fig. 5a); a gene that codes for *Zinc finger CCCH domain-containing protein* (Table 2) showed 4 splice variants in chromosome 3B, while one variant contained *Au* SINE within its transcript (Fig. 5b); a *Putative WRKY transcription factor* (Table 2) showed 2 splice variants in chromosome 5B, one of them harbored *Au* SINE within its transcript (Fig. 5c); and a *Transcription initiation factor* (Table 2) showed three splice variants in chromosome 7B, one of them contained *Au* SINE within its transcript (Fig. 5d). Figure S4 shows genes whereas some of their variants contain an *Au* SINE insertion within an exon. For example, Figure S4a presents a gene coding for 3-deoxy-manno-octulosonate cytidylyltransferase, mitochondrial protein that two of its variants (1, 2) contain *Au* SINE insertion in their last exon. Detailed analysis of the coding regions (CDS) in each splice variant revealed that in most cases the CDS of *Au* SINE-containing transcripts is shorter than the regular transcript leading, if translated, to a shorter protein (Fig. 5). For example: the CDS of the three *Au* SINE*-*containing splice variants of *putative methyltransferase* (Fig. 5a) lead to predicted protein sizes of 433 aa, while the CDS of the regular transcript (Fig. 5a) leads to a predicted protein size of 600 aa; the size of the predicted protein of *Zinc finger CCCH domain-containing protein* can reach up to 435 aa in the regular transcript, while it is 386 aa in the *Au* SINE*-*containing transcript (Fig. 5b); and the size of the predicted *WRKY transcription factor* is 495, while 355 aa in the *Au* SINE*-*containing transcript (Fig. 5c). In some cases, *Au* SINE or part of it became part of the coding sequence. For example, in TRIAE_CS42_2BL_TGACv1_131783_AA0432150 gene (Table 2, Figure S4f), the coding sequence of variant 1 does not contain *Au* SINE, but the coding sequence of variant 2 starts in a start codon located within an *Au* SINE insertion. Another example is TRIAE_CS42_3B_TGACv1_224095_AA0792250 gene (Table 2, Figure S4i) in which the coding sequence of variants 4 + 5 do not contain *Au* SINE, while the coding sequence of variants 1 + 2 + 3 + 6 contain different parts of the *Au* SINE insertion in their coding sequence. In variants 3 + 6, the coding sequence starts inside the *Au* SINE insertion. These data clearly indicate that *Au* SINE-containing introns underwent retention/exonizaion and became part of the mature transcript of many protein-coding genes.

**Table 2:** *In-silico* analysis of *Au* SINE-containing transcripts

Gene (EnsemblPlants)^a^	Gene product^b^	Location^c^	Number of splice variants^d^	Number of splice variants containing Au SINE^e^
TRIAE_CS42_1AL_TGACv1_002495_AA0042400	Putative methyltransferase PMT7	1A	5	3
TRIAE_CS42_1AS_TGACv1_020149_AA0075200	Protein STIP1-like protein / Ankyrin	1A	3	3
TRIAE_CS42_1BL_TGACv1_030243_AA0083440	3-deoxy-manno-octulosonate cytidylyltransferase, mitochondrial	1B	3	2
TRIAE_CS42_1BS_TGACv1_051223_AA0178630	Methyl-CpG-binding domain protein 4	1B	9	3
TRIAE_CS42_2AL_TGACv1_094480_AA0298520	Heterogeneous nuclear ribonucleoprotein Q	2A	6	1
TRIAE_CS42_2AL_TGACv1_095668_AA0313540	Cysteine proteinases superfamily protein	2A	3	1
TRIAE_CS42_2BL_TGACv1_130920_AA0419930	Serine/threonine-protein kinase	2B	3	1
TRIAE_CS42_2BL_TGACv1_131783_AA0432150	AMP-activated protein kinase, gamma regulatory subunit	2B	2	1
TRIAE_CS42_2BL_TGACv1_131823_AA0432620	SNARE associated Golgi protein family	2B	6	3
TRIAE_CS42_2BS_TGACv1_148673_AA0494490	Predicted membrane protein	2B	2	1
TRIAE_CS42_2DS_TGACv1_177434_AA0577040	Predicted protein	2D	3	1
TRIAE_CS42_3AL_TGACv1_193715_AA0618280	4-coumarate–CoA ligase-like 9	3A	8	1
TRIAE_CS42_3AL_TGACv1_194196_AA0628350	Predicted protein	3A	2	2
TRIAE_CS42_3AS_TGACv1_211514_AA0690950	Retinol dehydrogenase 14	3A	3	1
TRIAE_CS42_3B_TGACv1_222217_AA0760010	Potassium transporter 5	3B	2	2
TRIAE_CS42_3B_TGACv1_224095_AA0792250	Vacuolar-processing enzyme	3B	6	3
TRIAE_CS42_3B_TGACv1_227320_AA0822830	Zinc finger CCCH domain-containing protein 12	3B	4	1
TRIAE_CS42_4AL_TGACv1_288915_AA0961300	Protein kinase superfamily protein	4A	3	2
TRIAE_CS42_4AL_TGACv1_291111_AA0992310	Noncoding RNA	4A	2	1
TRIAE_CS42_4AS_TGACv1_306183_AA1003850	Periplasmic serine endoprotease DegP-like	4A	2	2
TRIAE_CS42_4BL_TGACv1_321946_AA1067110	Predicted protein	4B	3	2
TRIAE_CS42_4BS_TGACv1_328309_AA1086060	Protein CDC73 homolog	4B	3	1
TRIAE_CS42_4BS_TGACv1_328640_AA1091470	Predicted protein	4B	4	2
TRIAE_CS42_4DL_TGACv1_343838_AA1140270	FAR-RED IMPAIRED RESPONSE 1-like	4D	2	2
TRIAE_CS42_5AL_TGACv1_374408_AA1199410	DExH-box ATP-dependent RNA helicase DExH16, mitochondrial	5A	3	1
TRIAE_CS42_5AL_TGACv1_374413_AA1199550	Disease resistance RPP8-like protein 3	5A	2	2
TRIAE_CS42_5AL_TGACv1_375575_AA1223920	Non-coding RNA	5A	2	1
TRIAE_CS42_5BL_TGACv1_404363_AA1296950	Predicted protein	5B	3	2
TRIAE_CS42_5BL_TGACv1_406039_AA1339580	Carbamoyl-phosphate synthase small chain, chloroplastic	5B	4	2
TRIAE_CS42_5BL_TGACv1_407028_AA1352680	FBD-associated F-box protein	5B	2	2
TRIAE_CS42_5BL_TGACv1_407299_AA1355630	Signal recognition particle-related/SRP-related	5B	3	1
TRIAE_CS42_5BL_TGACv1_408403_AA1363260	Predicted protein	5B	9	1
TRIAE_CS42_5BS_TGACv1_424513_AA1390380	Putative WRKY transcription factor 3	5B	2	1
TRIAE_CS42_6AL_TGACv1_472758_AA1525700	U-box domain-containing protein 11	6A	3	1
TRIAE_CS42_6AS_TGACv1_485705_AA1550580	Predicted protein	6A	3	3
TRIAE_CS42_6BL_TGACv1_499355_AA1579140	Lysyl-tRNA synthetase	6B	4	2
TRIAE_CS42_6BL_TGACv1_501000_AA1612460	F-box/FBD/LRR-repeat protein	6B	4	1
TRIAE_CS42_6BS_TGACv1_514524_AA1660940	Predicted protein	6B	6	1
TRIAE_CS42_6BS_TGACv1_514925_AA1665920	Transcription termination factor MTERF8, chloroplastic-like	6B	2	2
TRIAE_CS42_7AL_TGACv1_557374_AA1780510	Polyadenylate-binding protein RBP45-like	7A	4	3
TRIAE_CS42_7AS_TGACv1_569582_AA1819670	Predicted protein	7A	3	1
TRIAE_CS42_7BL_TGACv1_577086_AA1865600	ELAV-like protein 1	7B	2	1
TRIAE_CS42_7BL_TGACv1_577812_AA1883950	Hydroxyproline O-galactosyltransferase HPGT1	7B	2	1
TRIAE_CS42_7BS_TGACv1_591848_AA1923570	Transcription initiation factor TFIID subunit 10	7B	3	1
TRIAE_CS42_7BS_TGACv1_593481_AA1951940	Putative clathrin assembly protein	7B	3	1
TRIAE_CS42_U_TGACv1_640735_AA2071780	Putative rust resistance kinase Lr10	NA	2	2
TRIAE_CS42_U_TGACv1_640941_AA2079970	disease resistance protein RPP13-like	NA	2	2
TRIAE_CS42_U_TGACv1_641735_AA2102860	Methionine S-methyltransferase	NA	2	2
TRIAE_CS42_U_TGACv1_641821_AA2105030	Putative Exocyst complex component 7	NA	2	1
TRIAE_CS42_U_TGACv1_643249_AA2129660	Mitochondrial inner membrane translocase complex, subunit Tim44-related protein	NA	2	1

^a^Gene accessions from *EnsemblPlants* (plants.ensembl.org/Triticum_aestivum)

^b^Based on gene annotation from *EnsemblPlants* with *e* value < 10^−10^.

^c^Chromosome location of the gene in *T. aestivum* genome. NA = not available

^d^The total number of splice variants for each gene detected from RNA-seq databases of *T. aestivum*

^e^The number of *Au* SINE-containing transcripts out of the total number of splice variants detected for each gene

## Conclusions

An updated genome sequence draft for *T. aestivum* revealed that bread wheat consists of ~ 100,000 genes (Clavijo et al. 2017) and that over 80% of its genome consist of TEs. Our estimation based on the current study and our previous reports (Ben-David et al. 2013; Yaakov et al. 2013a; Yaakov and Kashkush 2012) is that many wheat genes harbor at least one TE insertion, while most of the insertions are in intron regions. Plant TEs are considered one of the main components of the genome that are implicated in creating genetic variation among species. The insertion of TEs within genes might create allelic variation, and by such might impact gene expression. In this study, we provide data which led us to conclude that transposable elements, in this case a non-LTR retrotransposon termed *Au* SINE in wheat, might considerably impact gene structure and function by creating allelic variation and exonization in protein-coding genes. We have used very stringent parameters in the MAK software to retrieve *Au* SINE insertions from the updated RNA-seq database of *T. aestivum* (Clavijo et al. 2017); thus, the number of *Au* SINE-containing transcripts that were retrieved here (113 transcripts) might be an underestimate. We estimate that the intron retention of *Au* SINE might occur in hundreds of wheat genes. To this end, transcriptional interference induced by intronic retrotransposons might impact the transcription of large number of genes. Alternative splicing generates transcriptome variation that could lead to sub-functionalization of genes and speciation. Finally, because *Au* SINE is found in the entire plant kingdom (Deragon and Zhang 2006; Fawcett et al. 2006), we hypothesize based on our data that intron retention of *Au* SINE might be a general phenomenon in plants.

## Materials and methods

### Genomic data

In this study, three publicly available genome drafts were analyzed: (1) *T. urartu*, the donor of AA genome that was paired-end sequenced using whole-genome shotgun by Illumina [plants.ensembl.org/Aegilops_tauschii/Info/Index, (Ling et al. 2013)]. (2) *Ae. tauschii*, the donor of DD genome that was sequenced and assembled in the same way as *T. urartu* and the assembled scaffolds cover 83.4% of its genome with 90-fold depth reads. These reads combined with Roche-454 sequenced reads represent 97% of *Ae. tauschii* genome [plants.ensembl.org/Triticum_urartu/Info/Index, (Jia et al. 2013)]. (3) *T. aestivum*, the hexaploid bread wheat, which was published on June 2016 in *EnsemblPlants* [(Clavijo et al. 2017) pre.plants.ensembl.org/Triticum_aestivum/Info/Index]. This updated *T. aestivum* assembly was generated by The Genome Analysis Center in Norwich (TGACv1).

### Transcriptomic data

Here, we used the updated publicly available RNA-seq database of *T. aestivum* found in *Ensemblplants* [(Clavijo et al. 2017), plants.ensembl.org/info/website/ftp/index.html]. The library includes cDNA, CDS and ncRNA sequences that were used for annotation analysis in our study.

### Retrieval of *Au* SINE insertions

The sequences of *Au* SINE were retrieved from these genome drafts and transcriptome, using the MITE analysis kit (MAK) software [a standalone version was kindly provided by Guojun Yang, University of Toronto, (Janicki et al. 2011; Yang and Hall 2003a)]. The publicly available consensus sequence of the *Au* SINE family (GIRI database at http://www.girinst.org/repbase/update/browse.php) was used as an input (query sequence) in the MAK software and BLASTN was performed against the genomic drafts. For the retrieval of *Au* SINE-containing sequences from the genome drafts, we have used the MAK function “Member”, an *e* value of 10^−3^ and an end mismatch tolerance of 20 nucleotides. In addition, flanking sequences (500 bp from each end) were retrieved together with each one of the insertions, to characterize the insertion sites. A rice-specific MITE, called *mPing*, was used as a negative control in this analysis and no *mPing-*related sequences were retrieved in wheat. Redundant sequences were detected by BLAST + software (Camacho et al. 2009) using BLASTN function. We have compared sequences against themselves and excluded the paired element from each couple of sequences that were found to have a 100% identity (100% coverage with an *e* value of 0 and no gaps). The final output files were then edited using Textpad 7.4 ‘Regular Expression’ functions for cleaning excess data. It is important to mention that we have considered in this analysis truncated elements (at one of the terminal sequences) as being nearly intact elements.

### Insertion sites annotation

Annotation of *Au* SINE-flanking sequences was performed using the complementary-DNA (cDNA), coding sequences (CDS) and non-coding RNA (ncRNA) databases of *T. aestivum* (taken from *EnsemblPlants* at plants.ensembl.org/index.html). In addition, Transposable element consensus sequences from different plant genomes were also used as database in this annotation analysis (taken from ITMI at botserv2.uzh.ch/kelldata/trep-db/index.html). Annotation was performed using BLAST + standalone version 2.2.3 with an *e* value of 10^−10^. The merged 5′ and 3′ flanking sequences were used as query against the mentioned databases.

### Plant material, DNA and RNA extraction

In this study, we have used 21 accessions of seven *Triticum* and *Aegilops* species including the possible donors of AA (*T. urartu*, three accessions), BB (*Ae. speltoides*, and *Ae. searsii*, three accessions from each species), DD (*Ae. tauschii*, three accessions) genomes, and the allopolyploid species, *T. turgidum* (wild emmer and durum wheat, three accessions from each species) and *T. aestivum* (bread wheat, three accessions) seeds were kindly provided by Moshe Feldman, the Weizmann Institute of Science, Israel and the US Department of Agriculture (npgsweb.ars-grin.gov/gringlobal/search.aspx). Young leaves of ~ 4 weeks post germination plants were used for DNA (using GeneJET plant genomic DNA Purification Mini Kit, Thermo scientific) and RNA (using TRI reagent, Sigma) extractions. First strand cDNA was created using 5X All-In-One RT MasterMix (Applied Biological Material).

### Site-Specific PCR analysis

Insertional polymorphism of *Au* SINE was analyzed based on primers designed from flanking sequences (both sides) of *Au* SINE insertion. Primers were designed using PRIMER3 version 4.0.0 (bioinfo.ut.ee/primer3/) (see Table 1 for primer sequences). A full site includes a PCR product containing an *Au* SINE and flanking sequences, while an empty site lacks *Au* SINE (amplification of flanking sequences only). The reaction consisted of 12 µl ultrapure water (Biological Industries), 2 µl of 10× *Taq DNA polymerase* buffer (EURX), 2 µl of 25 mM MgCl_2_ (EURX), 0.8 µl of 2.5 mM dNTPs, 0.2 µl *Taq DNA polymerase* (5 U µl-1, EURX), 1 µl of each site-specific primer (50 ng µl^−1^) and 1 µl of template genomic DNA (approximately 50 ng µl^−1^). The PCR conditions for these reactions were 94 °C for 3 min, 30 cycles of 94 °C for 1 min, 58 °C for 1 min and 72 °C for 1 min, then 72 °C for 3 min. For sequence validation, PCR products were extracted from agarose gels using the QIAquick PCR Purification Kit (QIAGEN). Next, products were ligated into the pGEM-T easy vector (Promega, Madison, WI, USA) which was used for transformation into *E. coli* DH5α. Finally, for sequence validation, DNA products were sequenced by 3730 DNA Analyzer (Applied Biosystems, Foster City, CA, USA) at Ben-Gurion University, Israel.

### Expression analysis

Real-time quantitative RT-PCR (using 7500 Fast Real-Time PCR system, Applied Biosystems) was used to analyze the expression of genes harboring *Au* SINE in leaves of bread wheat (*T. aestivum*). For each gene, two primer pairs were designed; the first to amplify a regular transcript based on exon–exon junction amplification, and the second primer pair designed to amplify a chimeric transcript, if produced, consisting of *Au* SINE and flanking intron sequence. Primers were designed using the Primer Express v2.0 software and the PRIMER3 version 4.0.0 software (bioinfo.ut.ee/primer3/). Each reaction contained: 7.5 µl KAPA SYBR FAST qPCR Master Mix, 0.3 µl ROX Low 509—a reference dye for fluorescence normalization (KAPA BIOSYSTEMS), 1 µl forward primer (10 µM), 1 µl reverse primer (10 µM), 0.2 µl H_2_O (nuclease free water, Hylabs) and 5 µl or of cDNA template (50X dilution). The data were analyzed using the 7500 version 2.0.5 software (Applied Biosystems). The reaction conditions were 20 s at 95 °C, followed by 40 cycles of 3 s at 95 °C and 30 s at 60 °C. To differentiate specific PCR products from nonspecific ones, a melting curve was generated right after amplification by employing a 15 s incubation at 95 °C and a 1 min incubation at 60 °C, after which the temperature was raised by increments of 0.1 °C per sec until reaching 95 °C.

Data of each sample were received as Ct, threshold cycle of the PCR amplification reaching a certain level of fluorescence (Livak and Schmittgen 2001), normalized to the Ct of *ACTIN*, a known single copy gene used as an endogenous control. A comparative ${{\text{2}}^{ - \Delta \Delta {{\text{C}}_t}}}$ method was then used to determine the relative expression level of the two targets in each sample. First, each one of the normalized target expression levels in each sample was compared to the normalized target expression level of the reference sample, based on the following equation:

$$\Delta \Delta {C_{t({\text{test sample}})}}={\text{ }}{\left[ {{C_{t({\text{target}})}} - {\text{ }}{C_{t({\text{actin}})}}} \right]_{({\text{test sample}})}}-{\left[ {{C_{t({\text{target}})}} - {\text{ }}{C_{t({\text{actin}})}}} \right]_{({\text{reference sample}})}}.$$

Therefore, ${\text{RQ }}({\text{the relative expression level}}){\text{ }}={\text{ }}{({\text{2}}\, \times \,{\text{primer efficiency}})^{^{{ - \Delta \Delta {C_t}}}}}.$ Second, the two targets in each sample were compared to find their relative expression levels. Three technical replicates were used for each reaction to evaluate reproducibility. Standard deviations (SD) were calculated based on these three replications. Note that total RNA (not treated with reverse transcriptase) was used in RT-PCR reaction as a negative control for DNA contamination.

### *In-silico* analysis of *Au* SINE-containing transcripts

Au SINE-containing transcripts were further examined for all predicted variants of the same gene, as found in *Ensemblplants*. To validate these sequences are real transcripts and not genomic DNA sequences (due to contamination of the transcriptome), we compared these transcripts with the *T. aestivum* genome using BLASTN analysis to check whether we find full hits (meaning, genomic DNA sequence), or multiple partial hits for each sequence (meaning, mature RNA transcript). This was done by BLASTN algorithm with comparison of transcripts (query) to genomic database with an *e* value < 1e^−100^ (Table S3). This analysis showed whether each transcript had a full match (100% coverage) to sequence in the genomic database, or multiple partial matches to sequences in different locations of the genomic database. Using this analysis, we can eliminate transcripts that had full match and suspected to be genomic DNA or precursor RNA. To this end, all transcripts used here are mature RNA transcripts. The translated region of each transcript was determined by the CDS (coding sequence) as found in *Ensemblplants*. Each transcript containing an *Au* SINE insertion was traced back to its gene by transcript accession. All predicted variants of the same gene were examined in BLASTN analysis vs. *Au* SINE sequence and the specific location of insertion was determined in each variant.

#### **Author contribution statement**

DK: Generated the in-silico analysis data, analyzed results and manuscript preparation. CD: Generated the PCR and RT-PCR data, analyzed results. KK: (corresponding author). analyzed results, manuscript preparation and submission.

## Electronic supplementary material

Below is the link to the electronic supplementary material.

Supplementary material 1 (DOCX 4457 KB)

Supplementary material 2 (XLSX 160 KB)

Supplementary material 3 (DOCX 13 KB)

Supplementary material 4 (XLSX 110 KB)
