# Supplementary material for: Genome-wide analysis of a recently active retrotransposon, Au SINE, in wheat: content, distribution within subgenomes and chromosomes, and gene associations
Source: Plant Cell Rep. 2017 Nov 21;37(2):193–208. doi: 10.1007/s00299-017-2213-1 (PMC5787218; doi:10.1007/s00299-017-2213-1)
Supplement: Supplementary file 1 — Supplementary material 1 (DOCX 4457 KB) [file 299_2017_2213_MOESM1_ESM.docx]

**Supplemental Figures:**


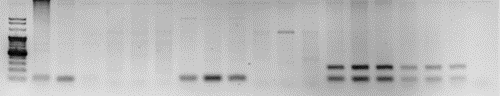


**b.**

1

2

3

1

2

3

1

2

3

1

2

3

1

2

3

1

2

3

AA

M

BB^2^

BB^1^

DD

AABB^2^

AABBDD

NC


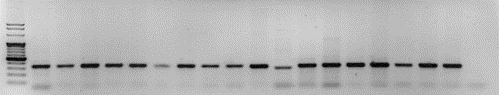


**a.**

1

2

3

1

2

3

1

2

1

2

3

1

2

1

2

1

2

3

M

BB^1^

BB^2^

AA

DD

AABB^2^

AABB^1^

AABBDD

NC


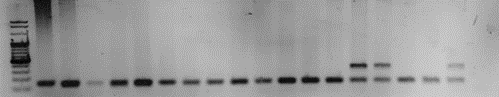


**c.**

1

2

3

1

2

3

1

2

3

1

2

3

1

2

3

1

2

3

AA

M

BB^2^

BB^1^

DD

AABB^2^

AABBDD

NC


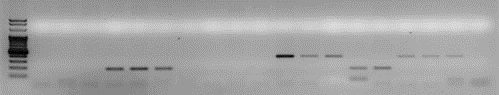


**d.**

M

BB^1^

BB^2^

AA

DD

AABB1

AABB^2^

AABBDD

NC

1

2

3

1

2

3

1

1

2

3

1

2

1

2

3

1

2

3

**e.**


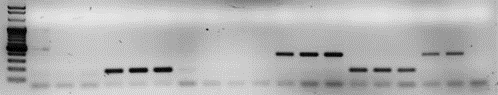


M

BB^1^

BB^2^

AA

DD

AABB^1^

AABB^2^

AABBDD

NC

1

2

3

1

2

3

1

1

2

3

1

2

1

2

3

1

2

3

**f.**


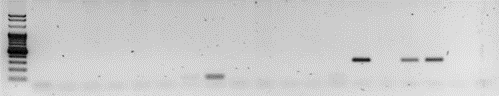


1

2

3

1

2

3

1

2

1

2

3

1

2

1

2

1

2

3

M

BB^1^

BB^2^

AA

DD

AABB^1^

AABB^2^

AABBDD

NC

**g.**


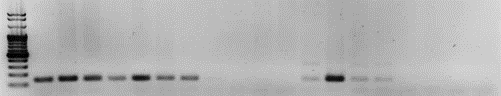


1

2

3

1

2

3

1

2

1

2

3

1

2

1

2

1

2

3

M

BB^1^

BB^2^

AA

DD

AABB^2^

AABB^1^

AABBDD

NC

**h.**


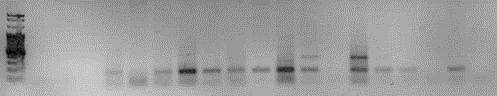


1

2

3

1

2

3

1

2

1

2

3

1

2

1

2

1

2

3

M

BB^1^

BB^2^

AA

DD

AABB^1^

AABB^2^

AABBDD

NC

**i.**


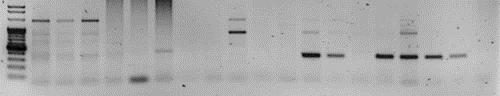


1

2

3

1

2

3

1

2

1

2

3

1

2

1

2

1

2

3

M

BB^1^

BB^2^

AA

DD

AABB^1^

AABB^2^

AABBDD

NC

**l.**


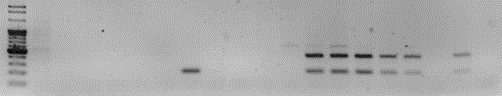


1

2

3

1

2

3

1

2

1

2

3

1

2

1

2

1

2

3

M

BB^1^

BB^2^

AA

DD

AABB^1^

AABB^2^

AABBDD

NC

**j.**


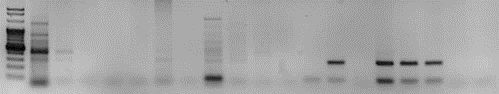


1

2

3

1

2

3

1

2

1

2

3

1

2

1

2

1

2

3

M

BB^1^

BB^2^

AA

DD

AABB^1^

AABB^2^

AABBDD

NC

**k.**


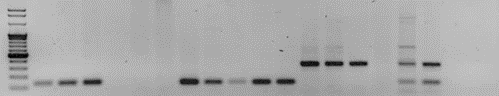


1

2

3

1

2

3

1

2

1

2

3

1

2

1

2

1

2

3

M

BB^1^

BB^2^

AA

DD

AABB^1^

AABB^2^

AABBDD

NC

**n.**

14

1

2

3

4

5

6

7

8

9

10

11

12

13

M


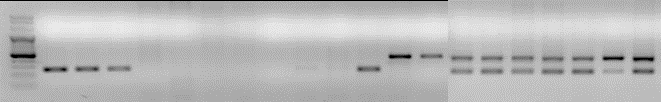


1

2

3

1

2

3

4

1

1

2

3

1

2

1

2

3

4

5

M

BB^1^

BB^2^

DD

AA

AABB^1^

AABBDD

6

**o.**


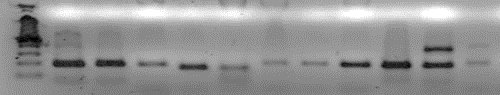


M

BB^1^

BB^2^

AA

DD

AABB^1^

1

2

3

1

2

3

1

2

3

1

2


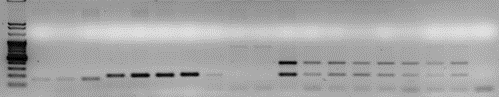


**m.**

M

BB^1^

BB^2^

AA

DD

AABB^1^

AABB^2^

AABBDD

NC

1

2

3

1

2

3

1

1

2

3

1

2

1

2

3

1

2

3

**p.**


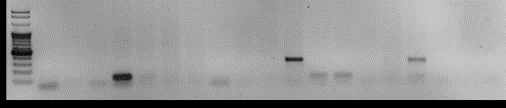


1

2

3

1

2

3

1

2

1

2

3

1

2

1

2

1

2

3

M

BB^1^

BB^2^

AA

DD

AABB1

AABB2

AABBDD

NC

**s.**


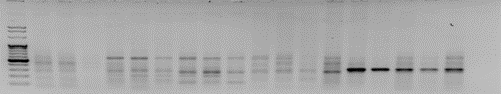


1

2

3

1

2

3

1

2

3

1

2

3

1

2

3

1

2

3

AA

M

BB^2^

BB^1^

DD

AABB^2^

AABBDD

NC

**q.**


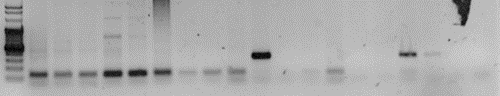


1

2

3

1

2

3

1

2

1

2

3

1

2

1

2

1

2

3

M

BB^1^

BB^2^

AA

DD

AABB^1^

AABB^2^

AABBDD

NC

**r.**


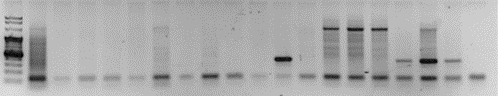


1

2

3

1

2

3

1

2

1

2

3

1

2

1

2

1

2

3

M

BB^1^

BB^2^

AA

DD

AABB^2^

AABB^1^

AABBDD

NC

**t.**


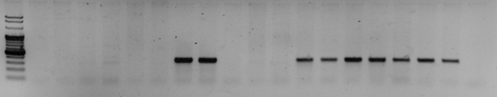


1

2

3

1

2

3

1

2

1

2

3

1

2

1

2

1

2

3

M

BB^1^

BB^2^

AA

DD

AABB^2^

AABB^1^

AABBDD

NC

**u.**


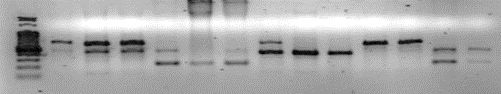


1

2

3

1

2

3

1

2

3

1

2

1

2

M

BB^1^

BB^2^

AA

DD

AABB^1^

**v.**


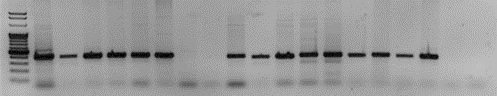


1

2

3

1

2

3

1

2

1

2

3

1

2

1

2

1

2

3

M

BB^1^

BB^2^

AA

DD

AABB^1^

AABB^2^

AABBDD

NC

**w.**


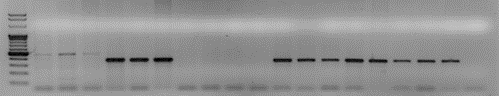


M

BB^1^

BB^2^

AA

DD

AABB^1^

AABB^2^

AABBDD

NC

1

2

3

1

2

3

1

1

2

3

1

2

1

2

3

1

2

3


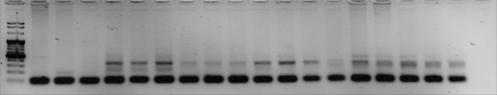


**x.**

1

2

3

1

2

3

1

2

3

1

2

3

1

2

3

1

2

3

AA

M

BB^2^

BB^1^

DD

AABB^2^

AABBDD

NC

**Figure S1.** Site-specific PCR analysis of insertional patterns of *Au* SINE in wheat genes (see Table 1). In each panel, upper arrow notes “full site”, while lower arrow notes an "empty site". “M” notes size marker, and “NC” notes negative control (ddH2O was used as template in PCR).
BB^1^ = *Ae. searsii*, BB^2^= *Ae. speltoides*, AA= *T. urartu*, DD= *Ae. tauschii*. AABB^1^= *T. durum*, AABB^2^= *T. diccocoides*, AABBDD= *T. aestivum*. Numbers above each lane represent the genomic replicates.
a). *Au* SINE monomorphic insertion in gene acc. TRIAE_CS42_2BL_TGACv1_129350_AA0379730. All accessions contain *Au*-SINE insertion within this gene and display a full site with the size of 354bp.
b). *Au*-SINE insertion in *T. diccocoides* and *T. aestivum* in gene acc. TRIAE_CS42_7BL_TGACv1_577920_AA1886220. Size of full site = 254bp.
c). *Au* SINE insertion in *T. diccocoides* and *T. aestivum* in gene acc. TRIAE_CS42_1BL_TGACv1_032021_AA0124300. Size of full site = 364bp, size of empty site = 198bp.

d). *Au* SINE insertion in *T. durum, T. diccocoides* and *T. aestivum* in gene acc. TRIAE_CS42_6BL_TGACv1_499645_AA1588080. Size of full site = 390bp, size of empty site = 209bp.

e). *Au* SINE insertion in genome *T. durum, T. diccocoides* and *T. aestivum* in gene acc. TRIAE_CS42_5BL_TGACv1_406235_AA1342580. Size of full site = 384bp, size of empty site = 204bp.

f). *Au* SINE insertion in genome *T. diccocoides* and *T. aestivum* in gene acc. TRIAE_CS42_2AL_TGACv1_093126_AA0272720. Size of full site = 305bp.

g). A unique *Au* SINE insertion in genome *T. durum* and *T. diccocoides* in gene acc. TRIAE_CS42_2BL_TGACv1_130367_AA0409600. Size of full site = 329bp, size of empty site = 166bp.

h). *Au* SINE insertion to *T. durum, T. diccocoides* and *T. aestivum* in gene acc. TRIAE_CS42_3B_TGACv1_220590_AA0709880. Size of full site = 381bp, size of empty site = 213bp.

i). *Au* SINE insertion in *T. durum*, *T. diccocoides* and *T. aestivum* in gene acc. TRIAE_CS42_1BS_TGACv1_049553_AA0156720. Size of full site = 349bp.

j). *Au* SINE insertion in *T. durum*, *T. diccocoides* and *T. aestivum* in gene acc. TRIAE_CS42_2BS_TGACv1_146572_AA0468420. Size of full site = 303bp, size of empty site = 122bp.

k). *Au* SINE insertion in *T. durum*, *T. diccocoides* and *T. aestivum* in gene acc. TRIAE_CS42_2AL_TGACv1_096183_AA0317680. Size of full site = 339bp, size of empty site = 158bp.

l). *Au* SINE insertion in *T. durum*, *T. diccocoides* and *T. aestivum* in gene acc. TRIAE_CS42_2BL_TGACv1_129880_AA0398630. Size of full site = 389bp, size of empty site = 208bp.

m). *Au* SINE insertion in the *T. durum*, *T. diccocoides* and *T. aestivum* in gene acc. TRIAE_CS42_1BS_TGACv1_049809_AA0161910. Size of full site = 399bp, size of empty site = 230bp.

n). *Au* SINE insertion in *T. durum* and *T. aestivum* in gene acc. TRIAE_CS42_5BL_TGACv1_405351_AA1325650. Size of full site = 472bp, size of empty site = 291bp.
o). *Au* SINE insertion in *T. durum* in gene acc. TRIAE_CS42_5BL_TGACv1_404700_AA1308770. Size of full site = 402bp, size of empty site = 221bp.

p). *Au* SINE insertion in *Ae. tauschii* and *T. aestivum* in gene acc. TRIAE_CS42_2DS_TGACv1_177403_AA0575920. Size of full site = 328bp, size of empty site = 147bp.

q). A unique *Au* SINE insertion in genome *Ae. tauschii* and *T. aestivum* in gene acc. TRIAE_CS42_4DL_TGACv1_343519_AA1135850. Size of full site = 344bp, size of empty site = 162bp.

r). A unique *Au* SINE insertion in genome *Ae. tauschii* and *T. aestivum* in gene acc. TRIAE_CS42_6DL_TGACv1_526989_AA1696800. Size of full site = 390bp.
s). A unique *Au* SINE insertion in genome *T. urartu*, *T. diccocoides* and *T. aestivum* in gene acc. TRIAE_CS42_4AL_TGACv1_290382_AA0984800. Size of full site = 318bp, size of empty site = 137bp.

t). A unique *Au* SINE insertion in genome *T. urartu*, *T. durum,* *T. diccocoides* and *T. aestivum* in gene acc. TRIAE_CS42_2AL_TGACv1_093836_AA0287830. Size of full site = 373bp.

u). *Au* SINE insertion in genome *T. urartu* and *T. durum* in gene acc. TRIAE_CS42_3AS_TGACv1_211370_AA0689310. Size of full site = 432bp, size of empty site = 263bp.

v). *Au* SINE insertion in all accessions except *T. urartu* in gene acc. TRIAE_CS42_4DL_TGACv1_342534_AA1116030. Size of full site = 396bp.

w). *Au* SINE insertion in *Ae. speltoides*, *T. durum,* *T. diccocoides* and *T. aestivum* in gene acc. TRIAE_CS42_2BS_TGACv1_145935_AA0449980. Size of full site = 378bp.

x). *Au* SINE insertion in all accessions except *Ae. speltoides* in gene acc. TRIAE_CS42_4AL_TGACv1_288293_AA0943850. Size of full site = 310bp, size of empty site = 129bp.


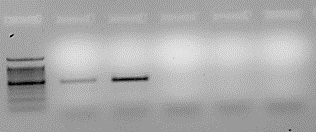

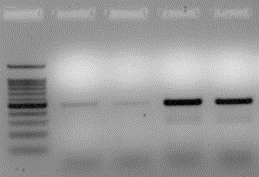


**M 1 2**

**M 1 2 3 4**

a.

b.

**Figure S.2. Validation of cDNA synthesis with actin control.** The cDNA generated from RNA of *T. aestivum* accessions was examined before using in real-time RT-PCR experiments for contamination of genomic DNA, using primers of two exons of the *Actin* gene (forward- GAAGCGCATATCCTTCGTAA; reverse- CCTTGTCTGTGACAATGGAA), giving different products for cDNA (494 bp) and for genomic DNA (874 bp). a. cDNA validation of *T. aestivum* accession 178383 (two independent reactions; 1 and 2) . b. cDNA validation of *T. aestivum* accession 377626 (4 independent reactions; 1-4). M notes the size marker. In both cases, only the 494 bp product was seen, indicating no DNA contamination.


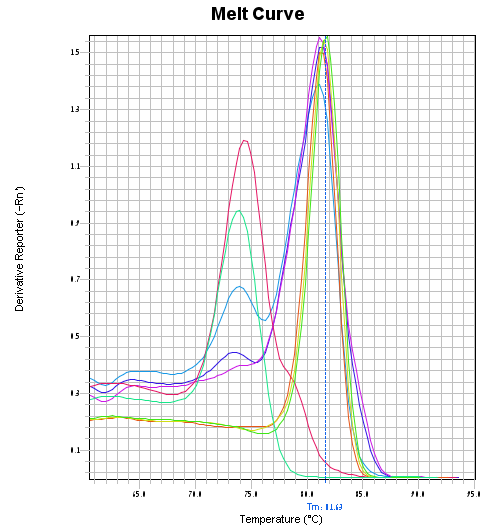

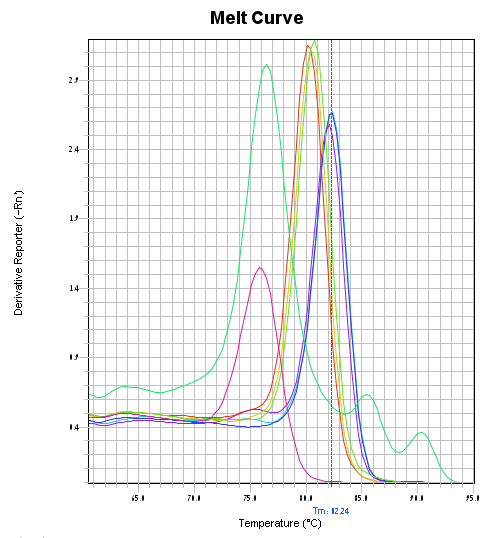

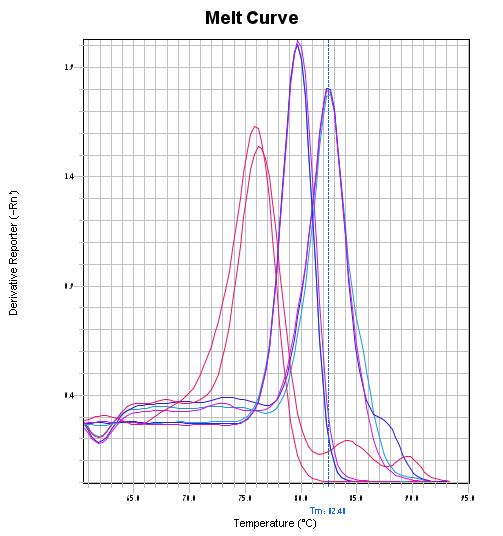

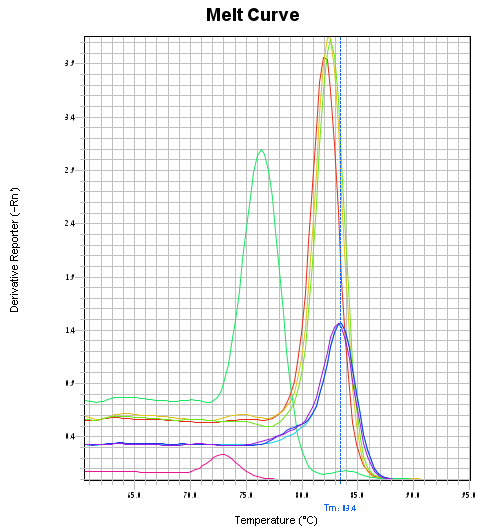


a.

b.

c.

d.

**Figure S.3. Identification of two different transcripts, a regular one and an *Au* SINE containing one.** Real Time qPCR melting curves results of the genes: (a) contig356155, (b) contig1161311 , (c) contig1151808 and (d) contig921670 that were found to have both transcripts; with *Au* SINE and without it. The reaction template for all genes was a mix of cDNA samples of two *T. aestivum* accessions (178383 and 377626). The production of the two transcripts emphasized by the two curves with two different melting temperatures.

**
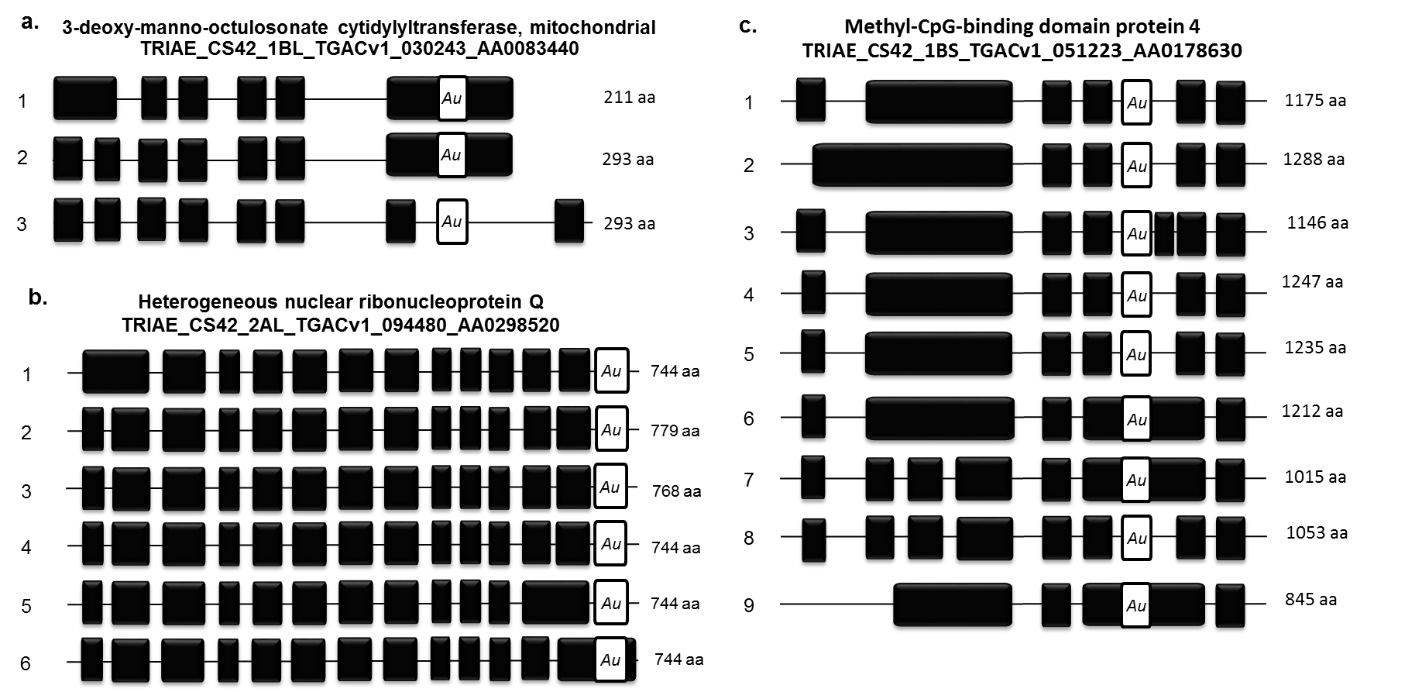
**

**
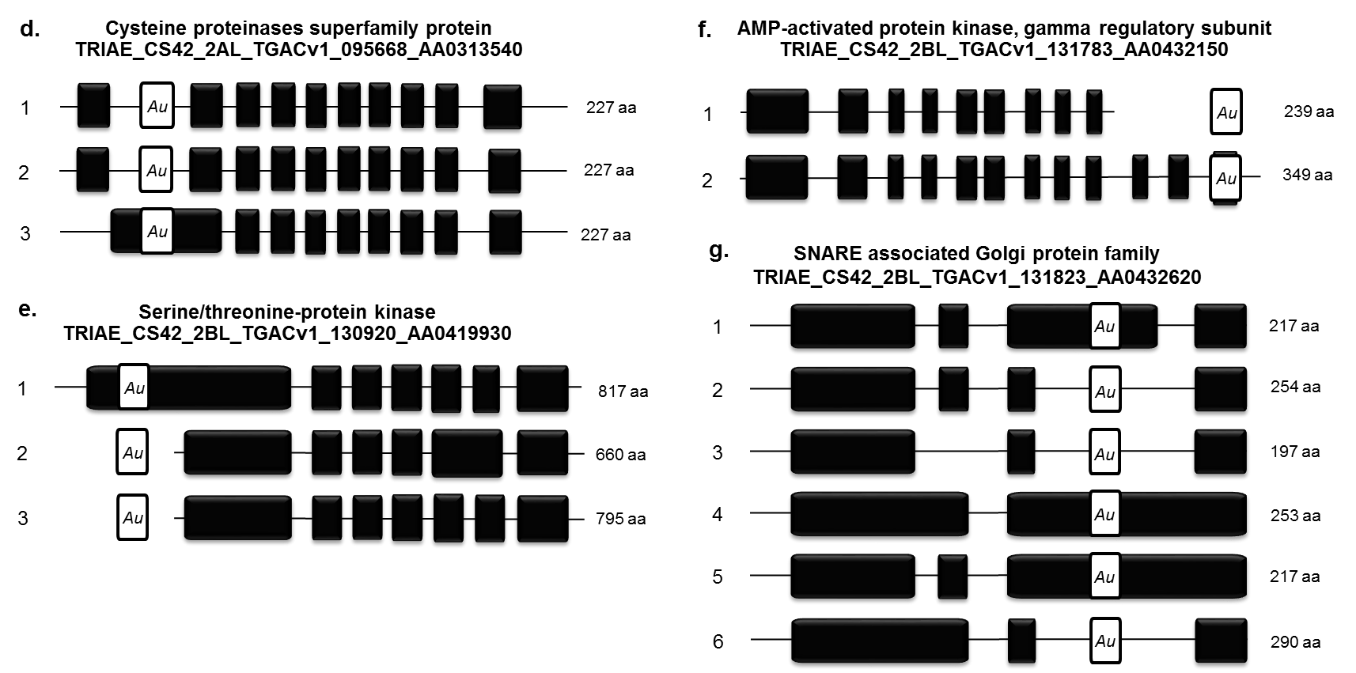
**

**
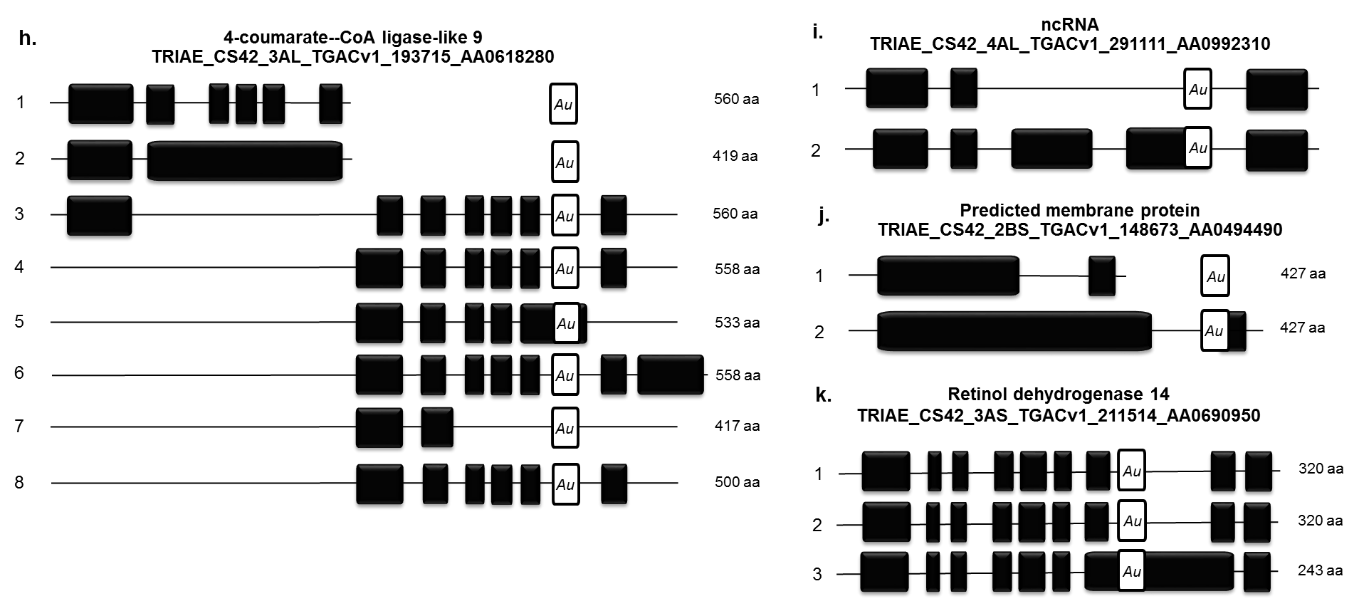

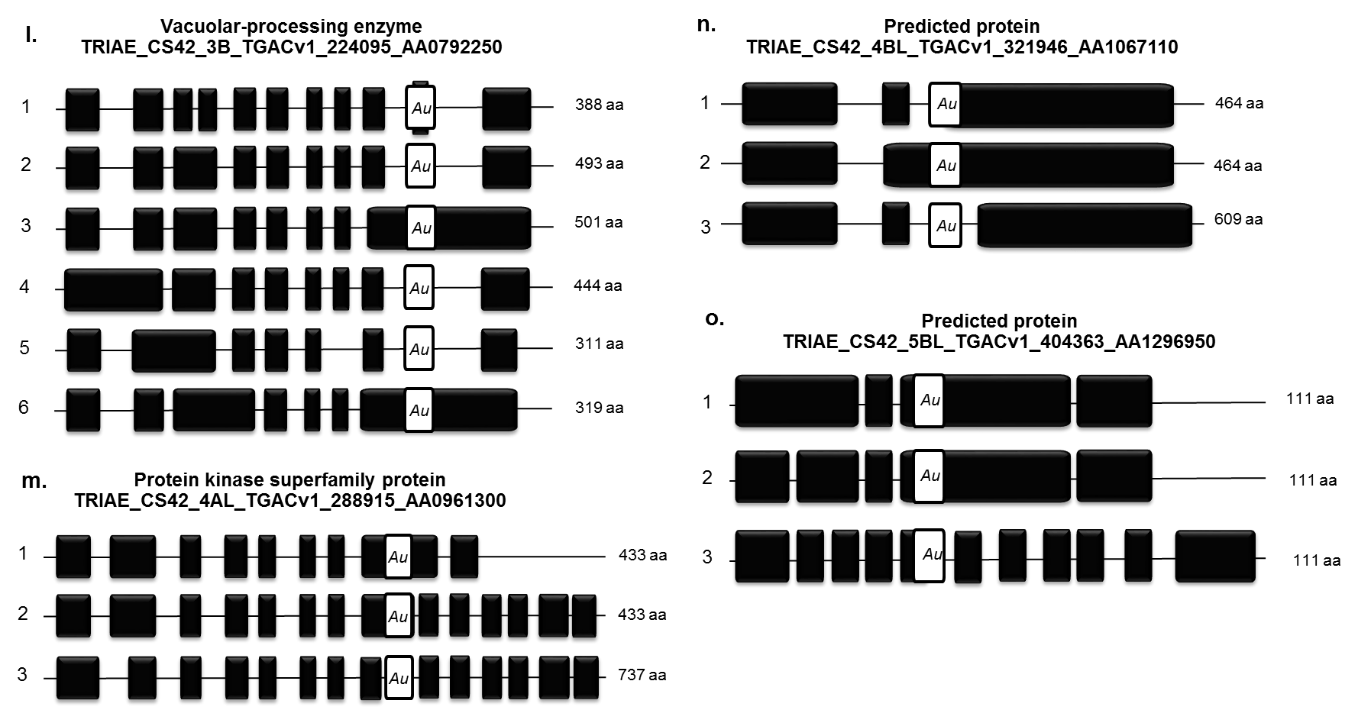
**


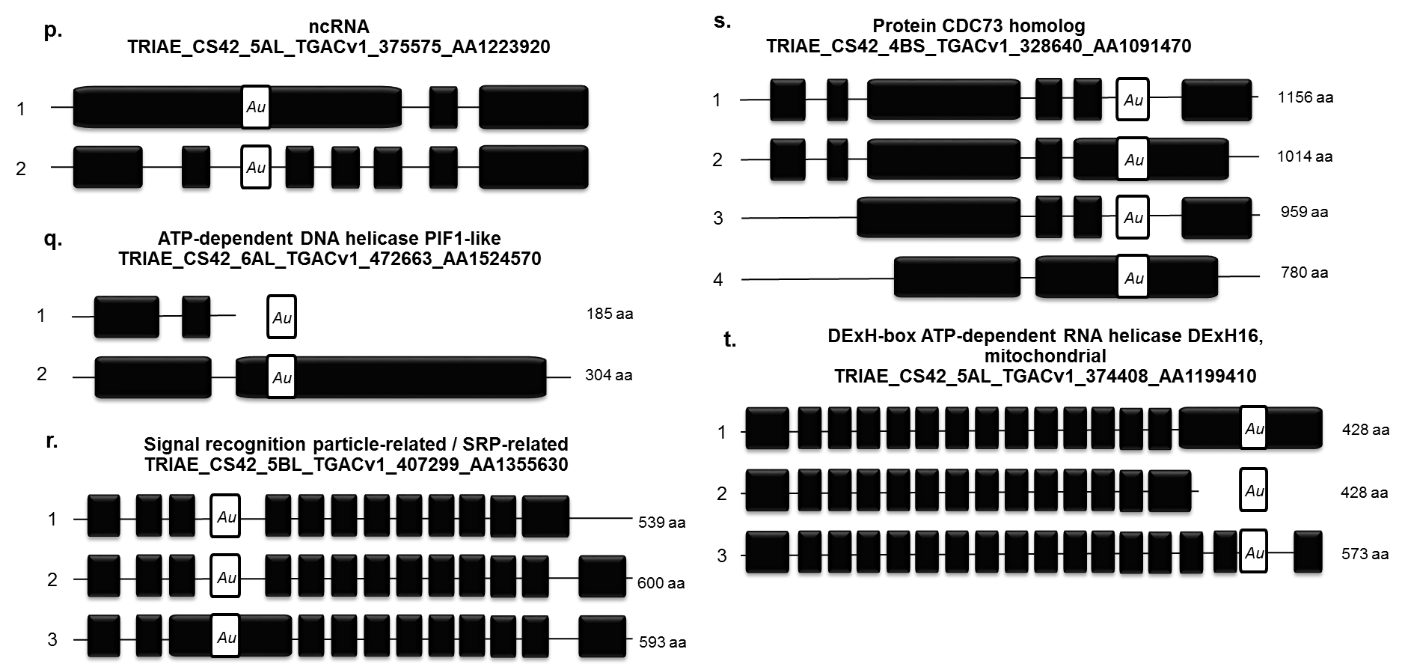

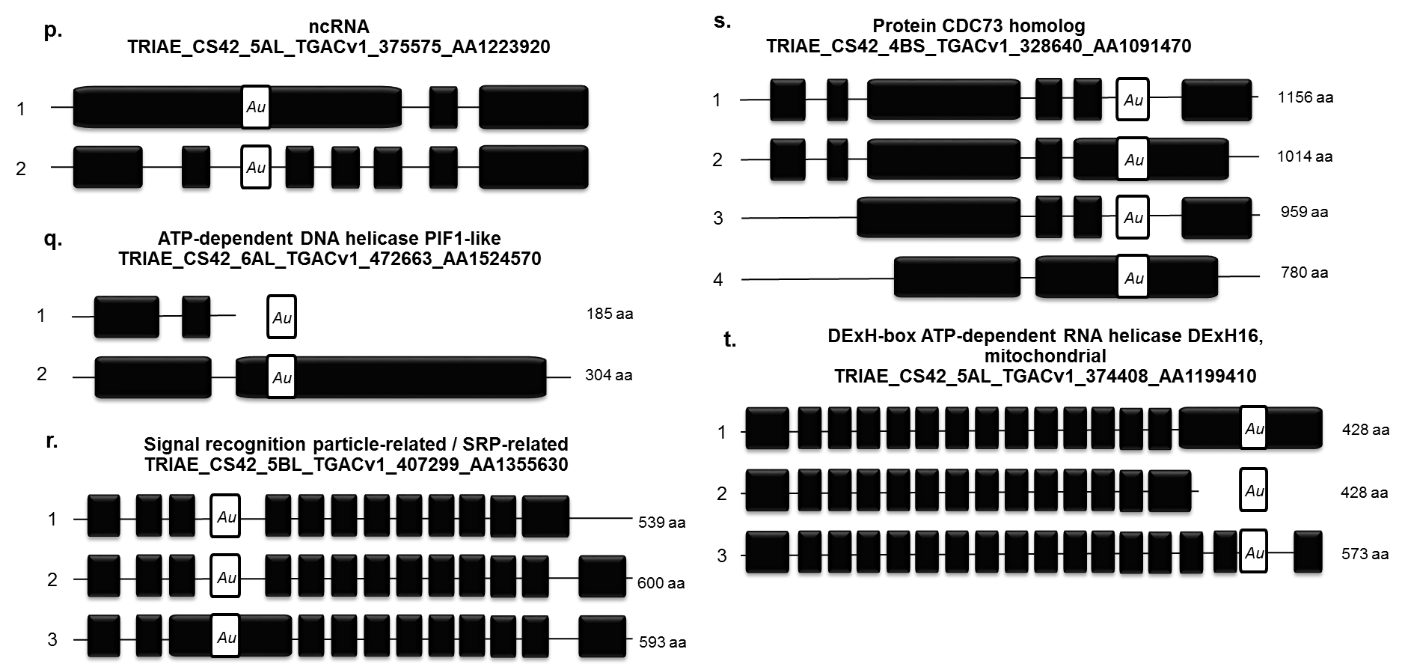

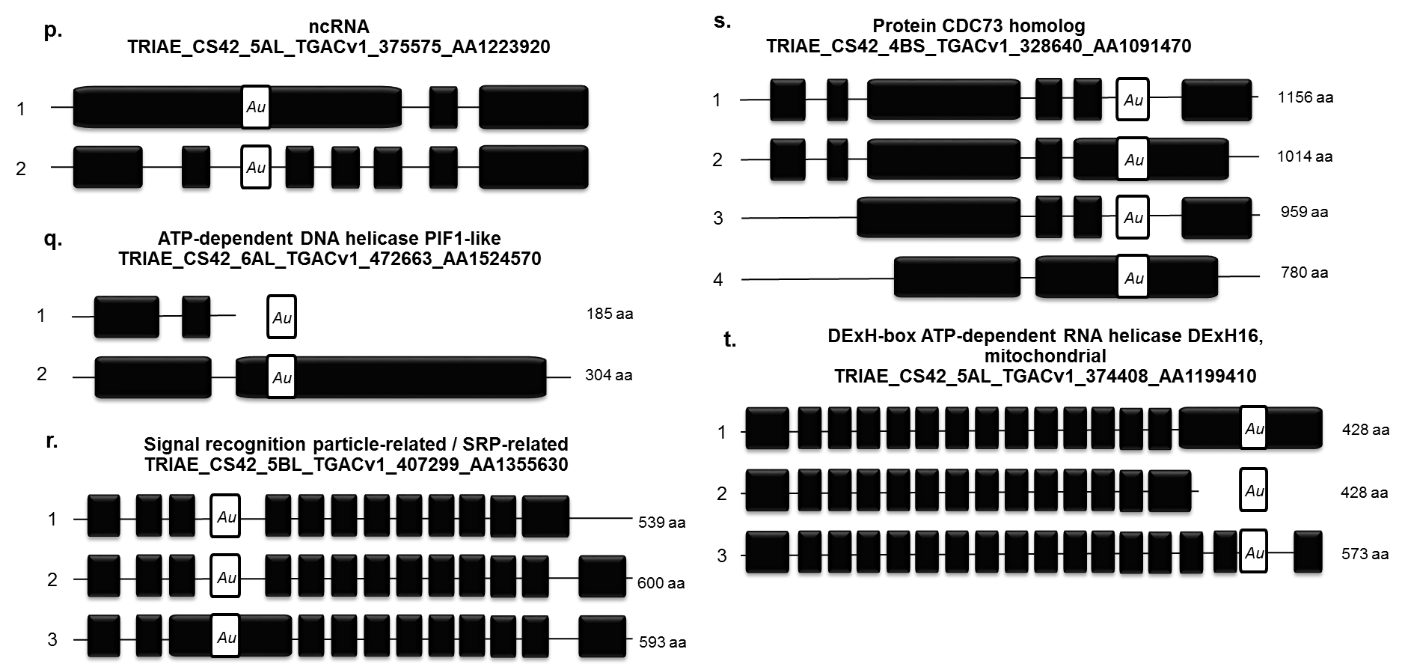


**
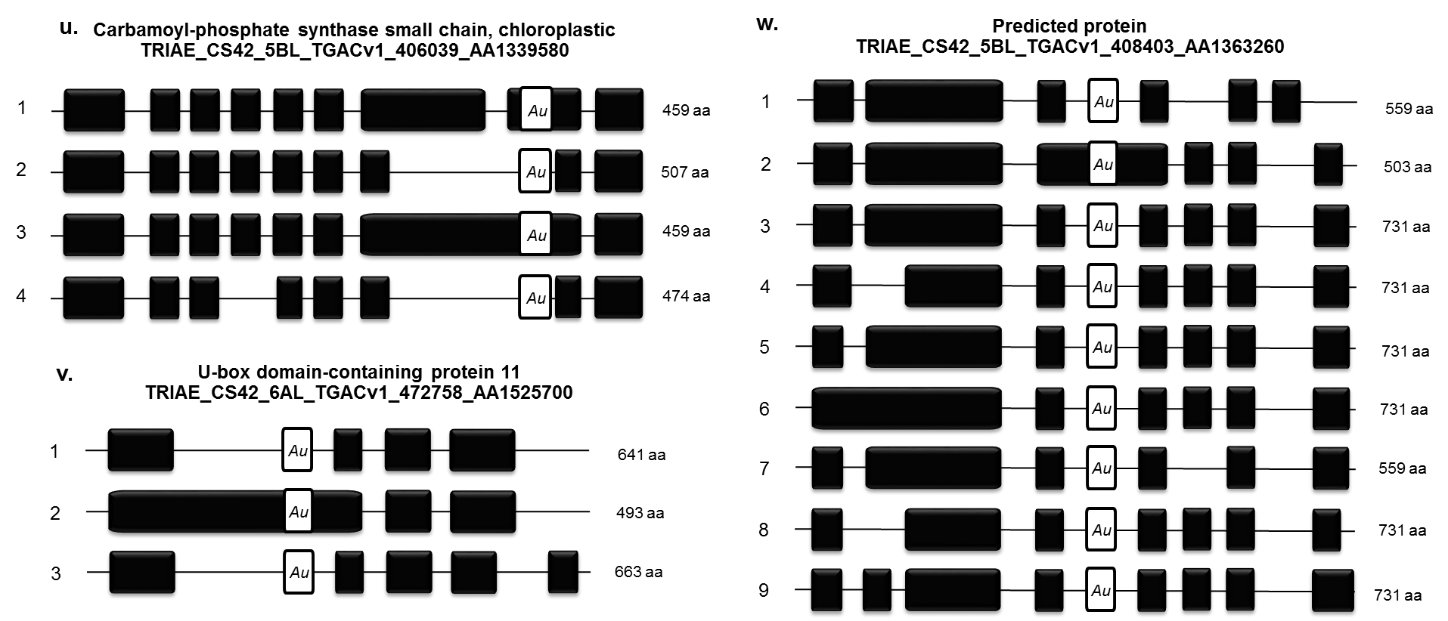
**

**
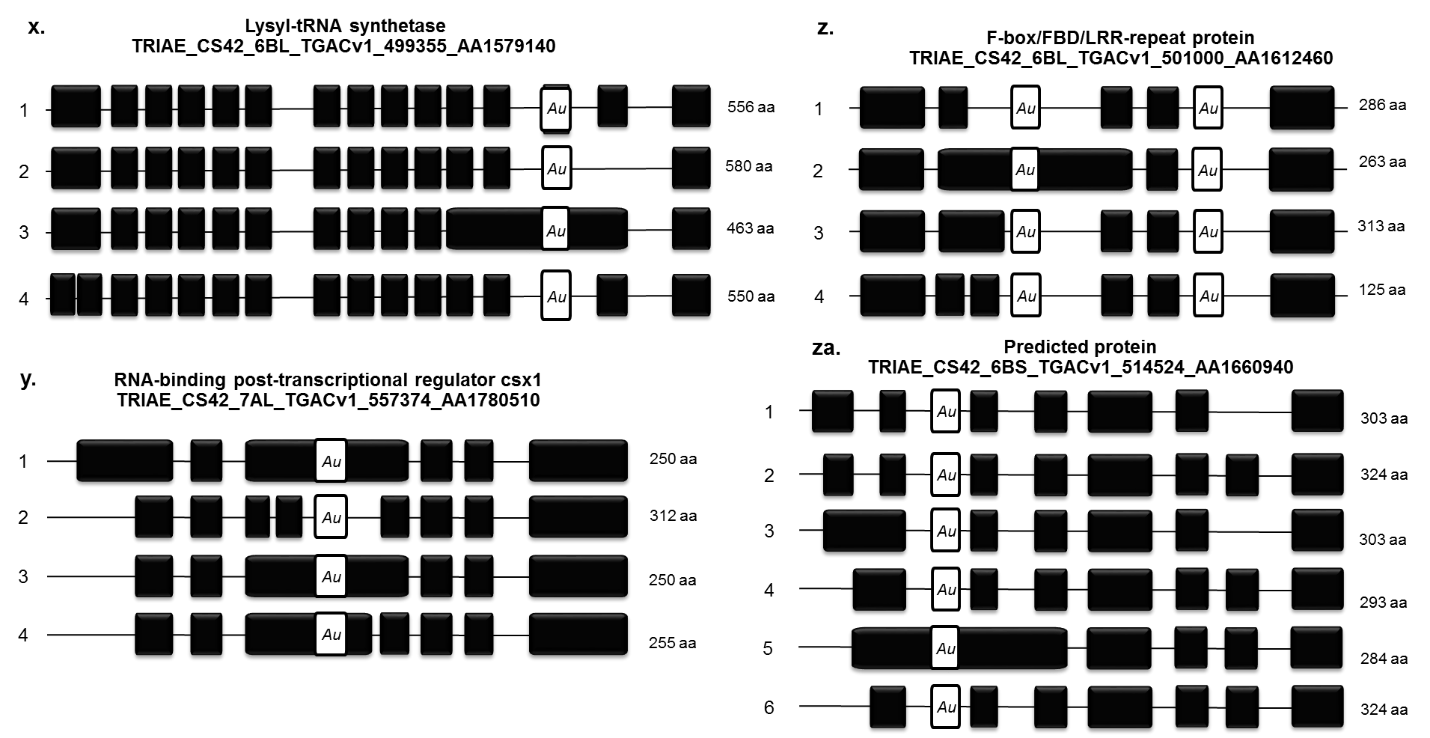
**

**
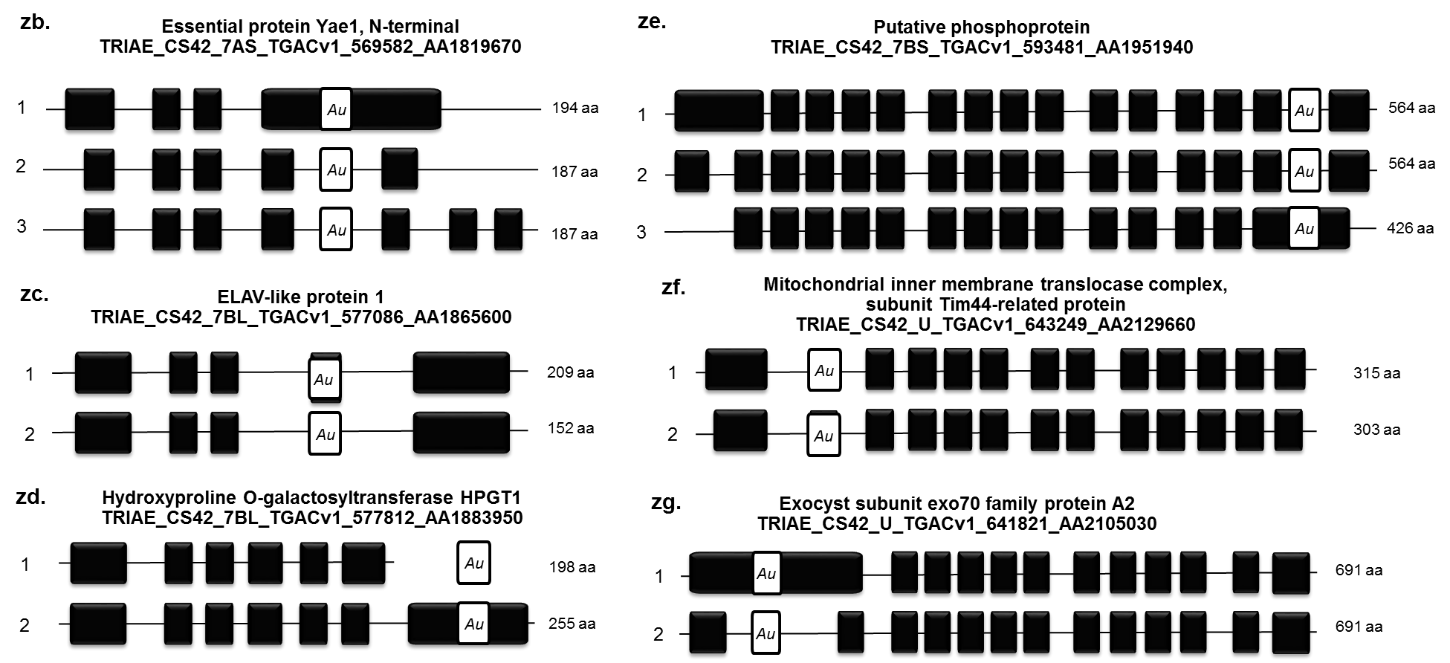
**

**
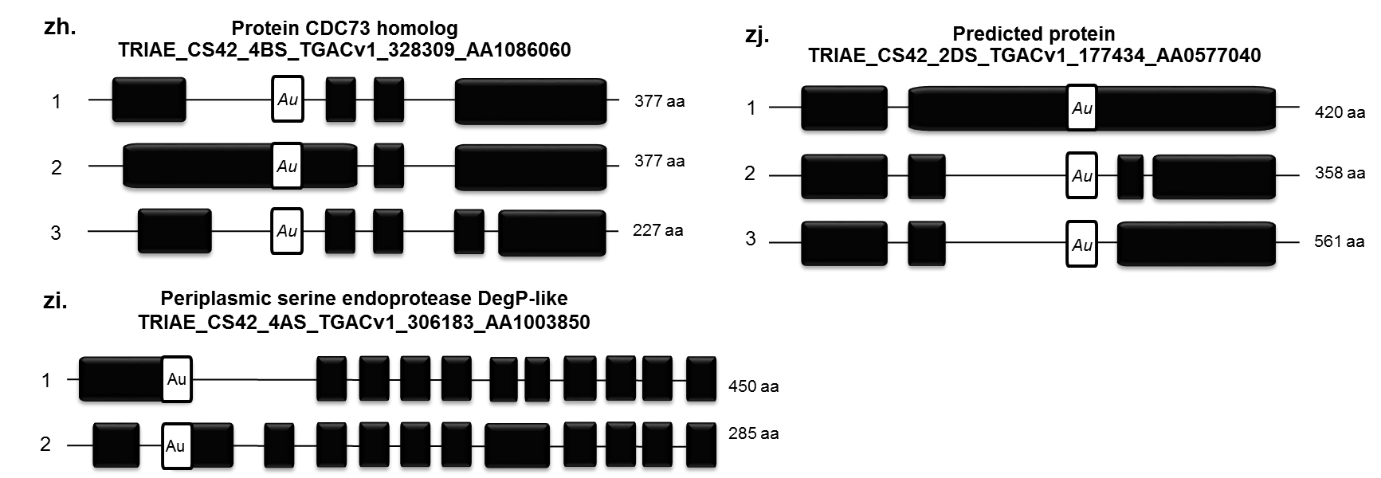
**

**Figure S.4.** Splice variants transcripts of *Au* SINE-harboring genes (a-zi). The name of the gene and *EnsenblPlants* accessions number are indicated on top. Black boxes note exons and lines note introns. Note that the mature transcripts consist of exons only, thus we kept here the intron regions in order to indicate the exact location of *Au* SINE (white boxes) in the mature transcript. The predicted protein for each splice variant is indicated on right of each panel.
