# Supplementary material for: Genome-wide analysis of a recently active retrotransposon, Au SINE, in wheat: content, distribution within subgenomes and chromosomes, and gene associations
Source: Plant Cell Rep. 2017 Nov 21;37(2):193–208. doi: 10.1007/s00299-017-2213-1 (PMC5787218; doi:10.1007/s00299-017-2213-1)
Supplement: Supplementary file 3 — Supplementary material 3 (DOCX 13 KB) [file 299_2017_2213_MOESM3_ESM.docx]

**Table S.2.** Primer sequences and their efficiency used in real time RT-PCR analysis

| **Gene accession number^1^** | **Amplification status^2^** | **Forward primer^3^** | **Reverse primer^3^** | **Primers efficiency^4^** |
| --- | --- | --- | --- | --- |
| TRIAE_CS42_1DL_TGACv1_061151_AA0187150 | - | CTTTTGATGGCACAGCACAT | CCAACAAGAACACACGCTTC | 95.71 |
| TRIAE_CS42_1DL_TGACv1_061151_AA0187150 | + | AGTTTGCGGTTCCAAGTCAC | TTCTGCAAAGAGGCTGTTCC | 100 |
| TRIAE_CS42_5BL_TGACv1_407697_AA1358910 | - | GTTGGTGCTTTCTCGCAGTT | AAATGGTTTGGGATGCAGAG | 100 |
| TRIAE_CS42_5BL_TGACv1_407697_AA1358910 | + | ACAAAGCAGAAGCATGAAAGG | GGGAAAGGCTGCGTACAATA | 100 |
| TRIAE_CS42_1BS_TGACv1_050314_AA0170550 | - | AGCAAGGCTGCTTATTGCAT | AGCCAGCCTAGTGAATCTGC | 100 |
| TRIAE_CS42_1BS_TGACv1_050314_AA0170550 | + | CCTGGACAGACTTGACAATCC | GGACTTGAACCCATGACCTC | 100 |
| TRIAE_CS42_4DL_TGACv1_342699_AA1119920 | - | TAATAAGTCCCATGGAGCACCG | CTGCAACACCACCATCCTTGT | 100 |
| TRIAE_CS42_4DL_TGACv1_342699_AA1119920 | + | GACTTGAACCCGTGACCTCATG | TGCTTGCGACACACCAAAAA | 100 |

^1^ Taken from *EnsemblPlants* database.

^2^ Primers were designed to amplify either the regular transcript or the *Au* SINE-containing transcript, while: -, no amplification; +, positive amplification.

^3^ Primer sequences used in this study (see Figure 3).

^4^ Primer efficiency parameter as was revealed by realtime RT-PCR analysis.
